# Unlocking the Dynamics of Human SPPL2a: First Atomistic Characterization of an Alzheimer’s-Linked Intramembrane Protease

**DOI:** 10.1021/acs.jcim.6c01556

**Published:** 2026-07-20

**Authors:** Sagnika Dutta, Mahender Kumar Singh, Budheswar Dehury

**Affiliations:** † Department of Bioinformatics, Manipal School of Life Sciences, 539679 Manipal Academy of Higher Education, Manipal, Udupi 576104, India; ‡ Data Science Laboratory, 29050 National Brain Research Centre, Gurgaon, Haryana 122052, India

## Abstract

The failure of γ-secretase inhibitors in clinical trials of Alzheimer’s disease (AD) has shifted the focus toward more selective targets, specifically SPPL2a (signal peptide-peptidase-like 2a), which is emerging as a critical therapeutic target in AD because of its role in processing TMEM106B. While recent cryo-EM structures have provided essential static snapshots, the dynamic mechanisms governing substrate entry and inhibitor recognition remain unresolved. Here, we present the first microsecond-time scale, all-atom molecular dynamics characterization of human SPPL2a in its apo and inhibitor-bound states, alongside a comparative analysis of the γ-secretase catalytic subunit, PS1. Our simulations reveal that SPPL2a is highly dynamic and undergoes an inhibitor-induced disorder-to-order transition in the TM6a region, where the helicity increases from 10% to 77%. We characterized the lateral gate dynamics of TM2, revealing that inhibitor binding fundamentally remodels the interhelical contact network and quenches conformational sampling. Moreover, we identified a unique inhibitor-stabilized lipid hotspot at Trp189 and demonstrated that SPPL2a utilizes a distributed polar network for binding energy, in contrast to the concentrated aspartate-driven affinity of PS1. These findings provide a quantitative atomistic blueprint of SPPL2a dynamics, offering a structural basis that can be exploited for the design of selectivity-guided therapeutics to bypass the clinical failures associated with nonspecific intramembrane protease inhibition.

## Graphical abstract

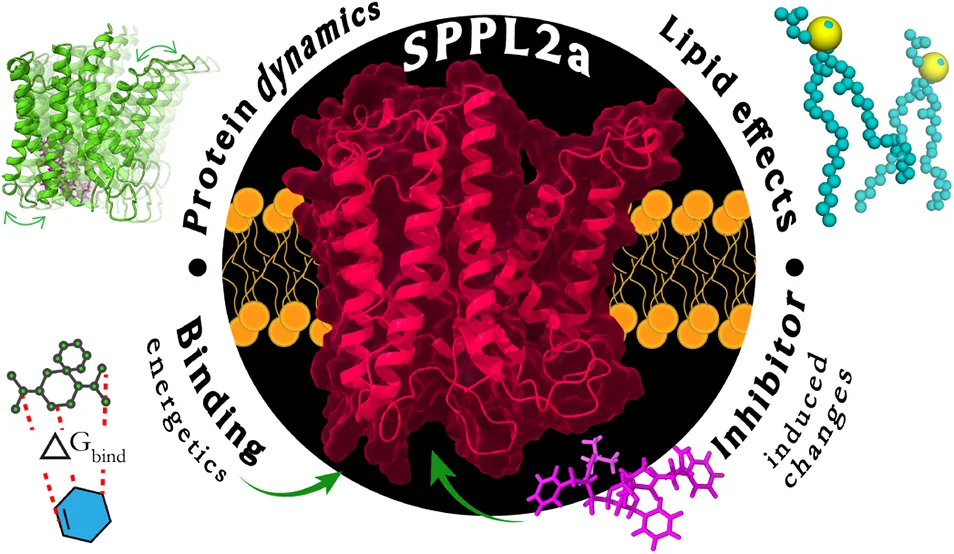

## Introduction

The hydrophobic core of the lipid bilayer represents one of the most chemically inhospitable environments in the cell for water-mediated reactions, yet what if you were told peptide bond hydrolysis, a reaction that requires precisely positioned water molecules and a nucleophilic attack can occur in this region? A seemingly unattainable task but exactly what a class of enzymes called intramembrane-cleaving proteases (i-CLiPs) perform within the lipid bilayer. RIP relies on i-CLiPs to execute signaling events, where the cascade begins with ectodomain shedding, resulting in the cutting of the extracellular segment of a transmembrane protein. Afterward, the i-CLiPs process the remaining membrane-bound fragment. i-CLiPs are ubiquitous in life and facilitate critical cellular functions, such as signal transduction and protein homeostasis.^1,2^ The roots of these enzymes could be traced back to the first account of intramembrane proteolysis in 1997—a study that revealed that site-2 protease (S2P) targets sterol regulatory element-binding proteins (SREBPs), cleaving them within the transmembrane.^3^ These enzymes are divided into four classes on the basis of their catalytic groups, all of which remain embedded in the lipid region: (a) zinc metalloproteases, (b) serine proteases, (c) glutamyl proteases, and (d) aspartyl proteases.^4^ Moreover, each of the classes has been implicated in various diseases, indicating that they are important for study.^5^

Among these, aspartyl proteases are primarily represented by Presenilins (PSEN/PS) and the signal peptide-peptidase (SPP)/signal peptide-peptidase-like (SPPL) family, both of which are nine-transmembrane segments containing a GxGD motif. However, they are distinguished by their functional organization; SPP functions independently, but PSEN is only catalytically active as a part of the γ-secretase multimeric complex.^6,7^ γ-secretase is known to cleave approximately 150 substrates, including Notch and amyloid precursor protein (APP), while the number of substrates known to be cleaved by SPPL members is very low.^7,8^ Research has focused on γ-secretase and its catalytic unit, PSEN, because its APP processing functions as a component of the amyloid-beta (Aβ) cascade and because missense mutations in this gene lead to early onset Alzheimer’s disease (AD).^4^ Numerous γ-secretase inhibitors (GSIs) have been developed over the past decade, and despite potent Aβ reduction, failed clinical trials due to side effects arising from the simultaneous inhibition of Notch signaling and other essential substrates, which is a consequence of insufficient selectivity.^6^ Large-scale genome-wide association studies (GWASs) have identified polymorphisms at the SPPL2A locus as significant risk factors for AD, and experimental evidence implicating SPPL2b in AD progression suggests that the SPPL family of proteases represents new avenues for exploration.^9–11^

The SPPL proteases were originally characterized by their structural homology to PSEN. They possessed an inverted topology to the latter, dictating the difference in their substrate cleavage, as they cleaved type II transmembrane segments with a cytosolic N-terminus, whereas γ-secretase targeted type I membrane proteins.^12^ The mammalian SPPL2a family consists of four members—SPPL2a, SPPL2b, SPPL2c, and SPPL3—which all share membrane topology. Among these, the most conserved member, SPPL3, is localized in Golgi bodies, where it maintains the equilibrium of protein glycan modifications. SPPL2c is localized in the endoplasmic reticulum and is present only in developing male germ cells, in stark contrast to the other members, which are more ubiquitous, where it supports acrosome integrity. SPPL2b is located mainly in the plasma membrane of neurons and glial cells, and SPPL2a is located in lysosomes and late endosomes. They play overlapping roles in the processing of substrates such as Dectin-1.^4,7^

SPPL2a processes substrates such as CD74 (the MHC class II invariant chain), the failure of which results in the loss of dendrite cells. Deficiency of SPPL2a was also observed to lead to Mendelian susceptibility to mycobacterial disease.^12,13^ SPPL2a also cleaves tumor necrosis factor α (TNFα) as well as TMEM106B, a protein associated with frontotemporal dementia (FTD) that is further implicated in AD pathogenesis.^4,10,14^ SPPL2a is defined by its C-terminal canonical tyrosine-based motif of the YXXø type, which is essential for its lysosomal sorting.^7^ For years, a significant gap in the characterization of the SPPL family existed because of the lack of structural data, which was further underscored by the contrasting progress in the structures of γ-secretase. This was recently resolved by Huang et al., who published the very first cryo-EM structure of human SPPL2a in ligand-free and L-685,458-bound states, establishing several important features, such as a preformed β-hairpin adjacent to the active site in the apo state, an element that in PSEN is induced only upon substrate engagement, suggesting that SPPL2a is primed for cleavage without requiring substrate-dependent rearrangement. Inhibitor binding also induces TM6a, a short helix near the active site region.^15^ While the cryo-EM structure provides a high-resolution map, it captures only a frozen snapshot of the molecular machinery that is always in motion. Molecular dynamics (MD) simulations precisely allow us to observe these dynamics at atomic resolution, yet no such study has been undertaken to elucidate the structural and conformational plasticity of SPPL2a alone and its inhibitor-bound states. Analogous studies of G protein-coupled receptors have demonstrated that ligand-induced conformational changes not only take place at the protein surface but also reach the membrane to modify lipid binding and residence times, thus establishing that the lipid bilayer actively participates in the conformational regulation of membrane-embedded enzymes.^16^

Consequently, elucidating the dynamic molecular mechanism of SPPL2a and resolving the complex conformational transitions and lipid-mediated regulatory mechanisms that occur within a native-like membrane environment become necessary. This is critical to further exploit its therapeutic potential in Alzheimer’s disease. In this study, we bridge this gap by deploying state-of-the-art, all-atom molecular dynamics (MD) simulations of human SPPL2a embedded in a POPC lipid bilayer at a microsecond time scale across three functional states—apo SPPL2a (PDB: 9K92), L-685,458-bound SPPL2a (PDB: 9K93), and compound E-bound PS1 from γ-secretase (PDB: 9K95). This research thus establishes the first dynamic, atomic-resolution characterization of SPPL2a, providing a quantitative atomistic blueprint to facilitate future structure-based drug discovery.

## Materials and Methods

### Acquisition of Structures

The cryo-EM structures solved by Huang et al. were retrieved from the Protein Data Bank (PDB) and used as starting coordinates for all the simulations: apo SPPL2a (PDB: 9K92), L-685,458-bound SPPL2a (PDB: 9K93), and compound E-bound PS1 from human γ-secretase (PDB: 9K95).^15^ The missing residues from these structures were modeled using Modeller10v7.^17^ The catalytic dyad of the two SPPL2a systems was modeled in its fully deprotonated state. In contrast, the PS1 system was modeled with a monoprotonated catalytic dyad (protonated Asp257 and deprotonated Asp385). To assess protonation state sensitivity, an additional 1 μs simulation of apo SPPL2a with Asp351 monoprotonated was performed and analyzed as a sensitivity control.

### System Assembly and Membrane Embedding

The systems were embedded in a homogeneous 1-palmitoyl-2-oleoyl-sn-glycero-3-phosphocholine (POPC) lipid bilayer, consistent with prior MD studies on the homologous aspartyl protease γ-secretase,^18,19^ to mimic native like membrane environment using the CHARMM-GUI membrane builder module.^20^ The CHARMM36m force field^21^ was employed to ensure the optimization of protein–membrane interactions, followed by system solvation via the TIP3P water model.^22^ Ions were added at a physiological concentration of 0.15 M NaCl to neutralize the system charge and maintain ionic strength. The assembled simulation systems contained the following components: (a) 9K92—250 POPC molecules, 21,333 TIP3P water molecules, 57 Na^+^ and 68 Cl^–^ ions, a total of 102,950 atoms, a 94.6 × 94.6 × 111.5 Å periodic box; (b) 9K93—247 POPC molecules, 19,606 TIP3P water molecules, 52 Na^+^ and 63 Cl^–^ ions, a total of 97,478 atoms, a 96.3 × 96.3 × 101.4 Å periodic box; (c) 9K95—256 POPC molecules, 41,577 TIP3P water molecules, 116 Na^+^ and 113 Cl^–^ ions, a total of 180,090 atoms, and a 102.6 × 102.6 × 166.1 Å periodic box.

### Molecular Dynamics Simulation

To assess the dynamic behavior of the proteases in a lipid bilayer environment, all-atom molecular dynamics (MD) simulations were performed for each system in triplicate, yielding nine independent trajectories via GROMACS v2021.4,^23^ allowing assessment of reproducibility. Prior to production, each system underwent energy minimization using the steepest descent algorithm to resolve clashes. Afterward, equilibration was performed following the standard protocol, a single NVT step followed by five sequential NPT steps.^24^ Production simulations of 1 μs were performed for each system under isothermal–isobaric (NPT) conditions at 303.15 K and 1 bar. The temperature was regulated using the Nosé–Hoover thermostat,^25^ and the pressure was controlled via semi-isotropic coupling using the Parrinello–Rahman barostat.^26^ A 2-femto-second integration time step was used, and the LINCS algorithm^27^ was utilized to constrain the hydrogen-containing bonds. The particle–mesh Ewald (PME) method was used for electrostatic calculations with a 1.2 nm cutoff, while van der Waals interactions were gradually truncated between 1.0 and 1.2 nm. All the systems were simulated under periodic boundary conditions. Simulation settings and parameters were implemented following the protocols established in our earlier research.^28^

### Trajectory Analysis

The built-in tools of GROMACS were used for the postsimulation analyses. Global structural metrics such as the backbone root-mean-square deviation (RMSD), radius of gyration (R*_g_*), and per-residue root-mean-square fluctuation (RMSF) of Cα atoms were computed using *gmx rms*, *gmx gyrate*, and *gmx rmsf*, respectively. The solvent-accessible surface area (SASA) and total number of hydrogen bonds in the system were calculated using *gmx sasa* and *gmx hbond*, and the evolution of the secondary structure was also tracked frame-by-frame using DSSP as implemented in *gmx dssp*. Afterward, trajectory visualization was performed in PyMOL.^29^ After which, electrostatic surface potential maps were calculated using the APBS plugin in PyMOL with default parameters on representative snapshots from each trajectory. Intramolecular hydrogen bonds were analyzed using MDAnalysis v2.10.0 ^30^ with the following geometric criteria: a donor–acceptor distance ≤ 3.5 Å and a donor–hydrogen–acceptor angle ≥ 150°. Occupancy was calculated as the fraction of trajectory frames in which each hydrogen bond was satisfied across the full 1 μs production trajectory.

### Principal Component Analysis (PCA) and Dynamic Cross-Correlation Matrix Analysis (DCCM)

PCA was performed to characterize the dominant collective motions of each system across the trajectory. To eliminate structural noise from flexible termini, analysis was restricted to backbone atoms of truncated protein chains. To isolate functional gating dynamics from bulk translation, every frame was aligned to a referenced target using a Kabsch alignment performed exclusively on the stable transmembrane anchor helices (TM4, TM5, TM7, and TM8). The analysis was conducted independently for each system using MDAnalysis and scikit-learn^31^ to center their respective conformational basins. The first two principal components (PC1 and PC2) were used to project the conformational landscape and identify distinct clusters sampled during the simulation. Porcupine plots illustrating the directionality and amplitude of the PC1 motions were generated. In addition, the DCCM was computed using a custom Python implementation based on MDAnalysis and NumPy^32^ to identify correlated and anticorrelated residue pairs that define allosteric communication pathways within each system, focusing on key transmembrane regions, specifically TM2, TM6, TM6a, and TM9.

### Binding Free Energy Calculations

Absolute binding free energies (ΔG_bind_) were estimated using gmx_MMPBSA v1.5.0.3 ^33^ on the last 200 frames from the final 200ns of each of the trajectories. The MM-PBSA method with dielectric constants of ε_in_ = 4.0 and ε_out_ = 80.0 at an ionic strength of 0.150 M was used. To identify energetic hotspots, per-residue interaction energy decomposition (*idecomp =* 1) was performed for residues in the protein–ligand interface.

$${\Delta G}_{\mathrm{bind}}={\Delta G}_{\mathrm{complex}}-{(\Delta G}_{\mathrm{receptor}}+{\Delta G}_{\mathrm{ligand}})$$

$${\Delta G}_{\mathrm{gas}}={\Delta G}_{\mathrm{vdw}}+{\Delta G}_{\mathrm{ele}}$$

$${\Delta G}_{\mathrm{solv}}={\Delta E}_{\mathrm{pb}}+{\Delta E}_{\mathrm{npolar}}+{\Delta E}_{\mathrm{disper}}$$

$${\Delta G}_{\mathrm{total}}={\Delta G}_{\mathrm{gas}}+{\Delta G}_{\mathrm{solv}}$$

The total binding energy was decomposed into van der Waals, electrostatic, and polar/nonpolar solvation contributions, with the latter estimated via the Poisson–Boltzmann and solvent-accessible volume (SAV) models. Entropic corrections were omitted, and the results are reported as the mean ± standard deviation.

### Membrane Dynamics

To evaluate the effects of the lipid membrane on the protease, the FATSLiM package^34^ was used to analyze membrane properties such as the area per lipid (APL) and bilayer thickness. Deuterium order parameters were computed to understand the fluidity gradient and generate density profiles for water, lipid headgroups, tails, and phosphate groups along the membrane normal (*z*-axis). Moreover, lipid interaction sites were identified with PyLipID^35^ using a dual-cutoff scheme with contact start and end distances of 0.55 and 0.35 nm, respectively, applied to POPC molecules across all three systems.

Plotting was performed with Python-based libraries—Matplotlib^36^ and ggplot2.^37^ All molecular graphics were generated on PyMOL and VMD, and further figure refinement and preparation were performed utilizing Adobe Illustrator.^38^

## Results and Discussion

### Structural Architecture, Membrane Topology, and Electrostatic Landscape

The SPP/SPPL family and the PS family represent two structurally convergent but topologically inverted groups of GxGD-type aspartyl intramembrane proteases, which share a conserved nine-transmembrane scaffold and a catalytic dyad embedded within the hydrophobic core of the lipid bilayer despite cleaving substrates of opposite membrane orientations^1^ (Figure 1A–B). On the one hand, the resolved cryo-EM structures reveal the horseshoe arrangement of the transmembrane helices with catalytic aspartates Asp351 and Asp412 embedded in TM6 and TM7, positioned toward the lumen of the cell membrane (Figure 1C). On the other hand, compared with TM6–TM7, PS1 organizes its TM helices in a topologically inverted but spatially equivalent manner, such as in terms of dyad spacing, PAL motif positioning, and β-hairpin location. Its catalytic aspartates, Asp257 and Asp385, are also located in TM6 and TM7, respectively, but are positioned toward the cytoplasm and not the lumen (Figure 1D). This inversion indicates that SPPL2a and PS1 recruit their respective substrates from opposite membrane faces, which is consistent with their complementary substrate specificities for type II and type I transmembrane proteins, respectively.^14^ These structural parallels, along with the shared dependence of both these proteases on a conserved PAL motif and β-hairpin adjacent to the active site, make PS1 one of the most informative structural references available for contextualizing SPPL2a dynamics. However, an important distinguishing factor is the antiparallel β-hairpin, which is preformed in SPPL2a, whereas in PS1, it is induced only upon substrate or ligand binding.^15^

**Figure 1:**
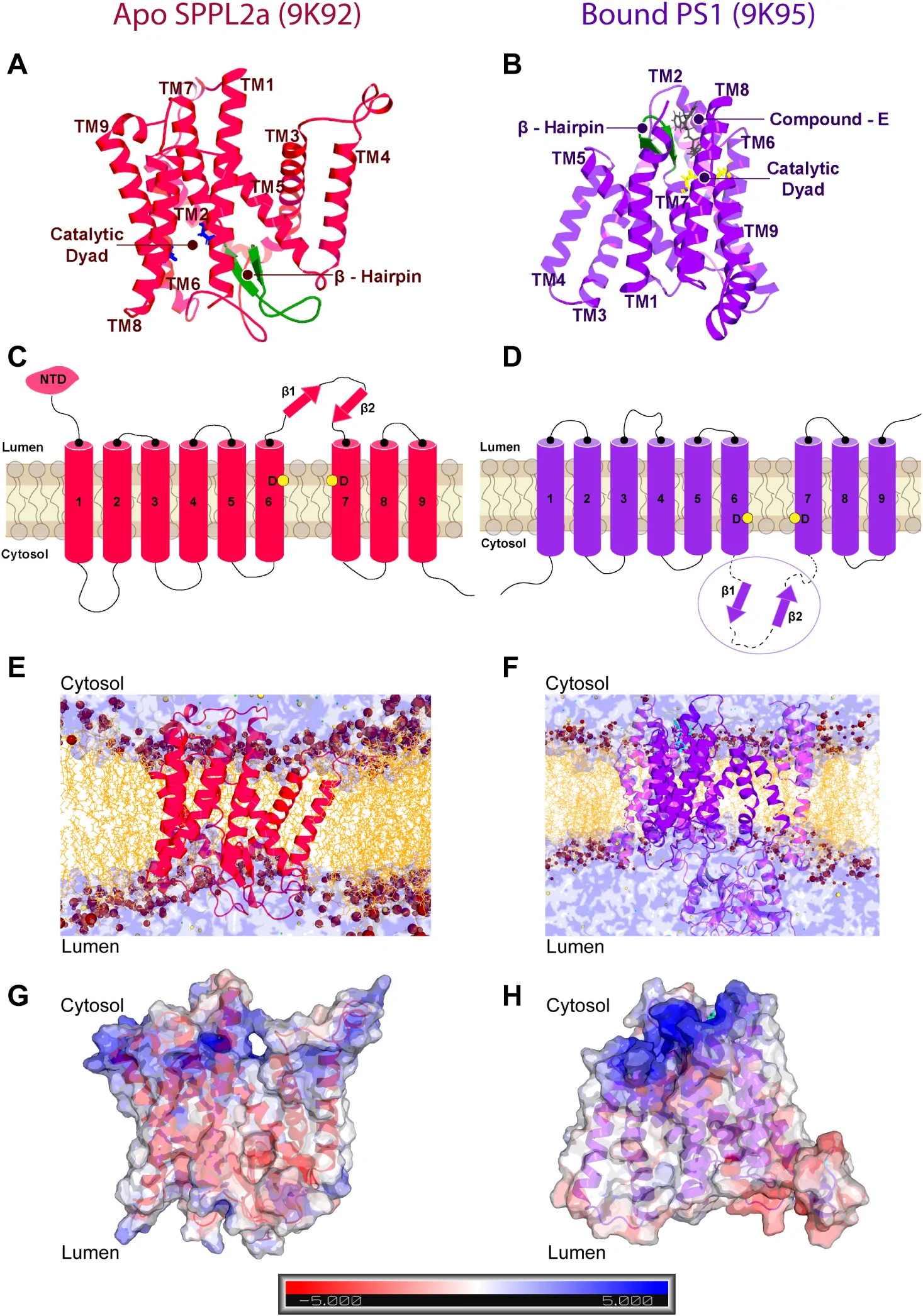
Structural architecture and environment of human SPPL2a and PS1. (A) Cartoon representation displaying the transmembrane helices, catalytic dyad and β-hairpin in SPPL2a. (B) Cartoon representation displaying the transmembrane helices, bound inhibitor, catalytic dyad and β-hairpin in PS1. (C–D) Topology diagrams illustrating the opposing membrane orientations of SPPL2a and PS1. (E) Human SPPL2a embedded in a POPC bilayer demonstrating the MD simulation environment. (F) Human γ-secretase embedded in a POPC bilayer (zoomed in on PS1 subunit) demonstrating the MD simulation environment. (G) Electrostatic surface potential map showing distributed electropositive potential near the cytosol in human SPPL2a. (H) Electrostatic surface potential map showing concentrated electropositive potential in the cytosolic region in bound PS1.

To investigate the impact of protein dynamics on these features, we ran 1 μs all-atom MD simulations of both SPPL2a and PS1 embedded in POPC lipid bilayers (Figure 1E–F). Analysis of the resulting electrostatic surface potential maps revealed that both SPPL2a and PS1 exhibit positive electrostatic potential in the cytosol-facing region, a finding noteworthy given that these proteases present their catalytic dyads and substrate-entry interfaces from opposite sides of the membrane but maintain equivalent positive surface characteristics in proximity to the cytosol (Figure 1G-H). This observation is in adherence with the positive-inside rule, which dictates that positively charged residues are selectively enriched in the cytoplasm facing regions as an evolutionarily conserved determinant of membrane protein stability.^39^ Another study conducted on topologically inverted tandem-repeat membrane proteins demonstrated that evolutionary constraints selectively conserve or introduce positively charged residues on whichever loops face the cytoplasm, regardless of the overall structural inversion, and that only a small number of such precisely placed charged residues are sufficient to satisfy cytoplasmic electrostatic requirements without disrupting the broader scaffold.^40^ This principle can be extended to the current observation as well, suggesting that cytosolic electropositive character is conserved in these aspartyl protease family members and reflects a shared requirement for membrane integration.

To confirm the stability and convergence of all three simulation systems prior to structural analysis, the backbone RMSD, radius of gyration (R_g_), and internal hydrogen bond counts were monitored across the full 1 μs trajectories. All three systems were simulated in triplicate and the replicate trajectories demonstrated comparable RMSD plateaus and highly correlated per-residue RMSF profiles across all systems, confirming the reproducibility of the conformational ensembles reported here (Figure S1). Apo SPPL2a reached a stable phase at approximately 896 ns, with an RMSD of 0.52 ± 0.02 nm, reflecting the broader conformational sampling available to a ligand-free system. Compared to apo SPPL2a, inhibitor-bound SPPL2a converged faster at approximately 801 ns and stabilized at an RMSD of 0.37 ± 0.01 nm, which is consistent with the inhibitor-induced restriction of backbone flexibility (Figure S2A). The full γ-secretase complex converged at 826 ns with an RMSD of 0.35 ± 0.03 nm, and the PS1 subunit isolated from the same trajectory converged with an RMSD of 0.172 ± 0.01 nm at approximately 598 ns (Figure S2B), reflecting the conformational restraint imposed by the surrounding subunits. The R_g_ values remained stable throughout the postconvergence phase across all the systems, with both apo and bound SPPL2a having an R_g_ of approximately 2.2 nm, γ-secretase having an R_g_ of approximately 4.4 nm and PS1 of approximately 2 nm (Figure S2C–D), and internal hydrogen bond counts showing no systematic drift (Figure S2E–F), confirming that all three systems achieved equilibrated, conformational ensembles.

### TM2 Lateral Gate Dynamics Reveal Inhibitor-Driven Conformational Changes and Cross-Helix Contact Remodeling

The TM2 helix forms the lateral gate, controlling access to the active site of SPPL2a. A breakthrough study by Huang et al. demonstrated that the TM2 helix and loop-1 are more mobile in the apo state than in the bound state and that TM2 was observed to have moved approximately 2.4 Å.^15^ The present MD simulation extends this static structural observation by resolving the full dynamic envelope of TM2 displacement in both states across the 1 μs time scale. To understand the gate behavior accurately, three interhelix distances were calculated: TM2–TM3 gate sealing, TM2–TM6 opening of the gate, and TM2–TM9 width. In the apo state, the gate sampled an open, dynamically fluctuating ensemble, with the width of TM2–TM9 exhibiting the greatest absolute displacement among the three metrics (Table 1). Upon L685,458 inhibitor binding, the distance between TM2 and TM9 decreased substantially along with an increase in the variance of all three metrics in comparison to the apo system, indicating that the inhibitor does not rigidly lock the gate but instead redistributes the conformational sampling to a closer configuration than the apo state does. This finding aligns with the observations of the static structure but reveals that this protein involves more than just simple rigidification. Notably, the TM2–TM6 distance modestly increases in the bound state, suggesting that the closure of the TM2–TM9 pore is accompanied by slight outward displacement of TM6, which might be due to the transition of TM6a from the coil to the helix. However, these simulations were conducted in the absence of a substrate, meaning that the lateral gate distance metrices describe the intrinsic conformations and inhibitor-driven changes as opposed to substrate-driven gating changes.

**Table 1:** Interhelix Distances (in Å) between the Transmembrane Helices Surrounding TM2 throughout the Simulation

Metric	System	Mean	Std. dev	Min.	Max.
TM2–TM3 (Gate Seal)	Apo SPPL2a	23.67	0.89	21.64	26.21
TM2–TM3	Bound SPPL2a	22.14	1.48	17.93	27.01
TM2–TM6 (Gate opening)	Apo SPPL2a	14.17	0.79	12.15	16.39
TM2–TM6	Bound SPPL2a	14.9	1.18	11.44	19.61
TM2–TM9 (Pore Width)	Apo SPPL2a	30.81	1.15	28.44	34.45
TM2–TM9	Bound SPPL2a	23.15	1.4	19.54	27.93

To establish the residue-level basis for these conformational changes, a comparative analysis of the contact maps between TM2 and its neighboring residues was conducted for both the apo and bound states across the full simulation trajectory (Figure 2A). Excluding the residue contacts present for structural stability, a total of 33 *de novo* contacts were identified in the bound state. Three contacts were maintained for more than 95% of the simulation—residue 350 in TM6 (99.9%), residue 368 in TM6a (97.6%), and residue 372 in TM7 (95.4%), with TM6–TM7 being the helices that harbor the catalytic aspartate dyad. This cross-linking of the lateral gate helix to the catalytic dyad containing helices provides a basis for inhibitor driven substrate entry suppression. This mechanism parallels the observation documented by Li et al. in PS1, where compound E binding simultaneously closed the lateral cleft and disabled the initial substrate docking site.^41^ Inhibitor binding also resulted in the dismantling of 9 contacts; most prominently, the protein–lipid interaction (represented as residue 497 in Figure 2) decreased from 78.1% occupancy in apo to zero upon binding. This finding indicates that L-685,458 binding does not simply shift the TM2 position but fundamentally remodels the interhelical contact architecture of the gate. This loss of the protein–lipid contact suggests that inhibitor binding drives gate closure partly through the physical displacement of a surrounding lipid, replacing the interaction with a protein–protein contact network, locking the helix into a new set of sustained interactions.

**Figure 2:**
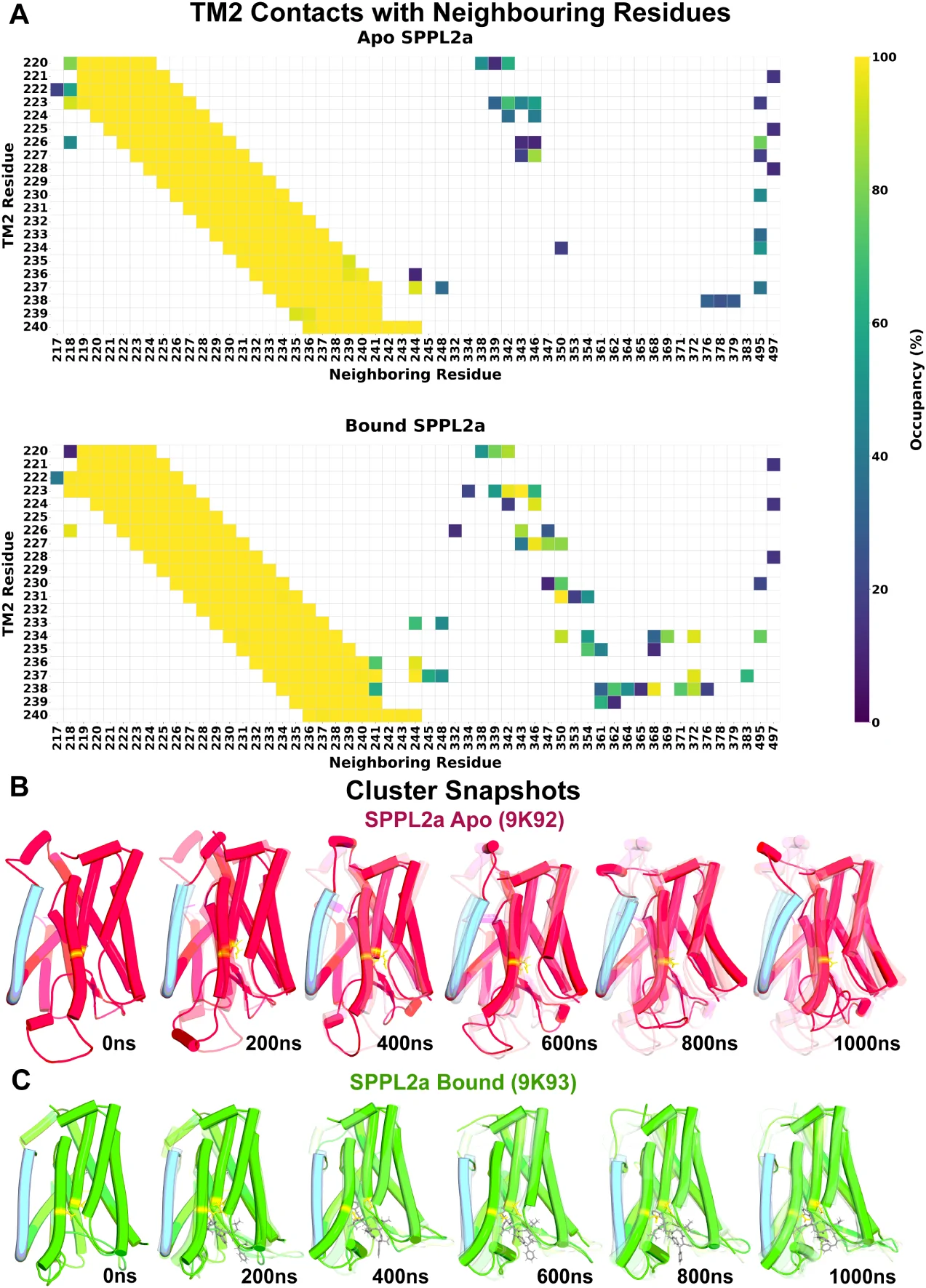
Inhibitor binding restricts TM2 gate mobility and reconfigures the interhelical contact network. (A) Contact frequency heatmap of TM2 with the neighboring residues across the 1 μs simulation, which transitions from dark purple to light yellow with increasing occupancy of the contact. (B) Representative cluster snapshots of apo SPPL2a at 200 ns intervals. (C) Representative cluster snapshots of inhibitor-bound SPPL2a at 200 ns intervals; TM2 is represented in light blue, the catalytic dyad in yellow and the inhibitor in gray.

Representative cluster centroid snapshots were extracted at 200 ns intervals across each of the trajectories, with TM2 highlighted in blue. In apo SPPL2a (Figure 2B), successive clusters clearly show displacement of TM2 along with the rest of the protein, demonstrating that the protein helix samples have a wide conformational envelope throughout the simulation with no stable resting geometry, whereas in the inhibitor-bound system, TM2 displacement between successive clusters is substantially diminished (Figure 2C). These findings corroborate the results obtained from the contact map to further establish that the degree of freedom of the TM2 gate is quenched upon binding of the inhibitor.

### Inhibitor-Induced Transition and Stabilization of the TM6a Helix in SPPL2a

In the apo state of SPPL2a, the TM6a region exists predominantly as a coil throughout the trajectory, with a mean helicity of only 10.36%, reflecting its intrinsically disordered nature when ligand-free. Upon L-685,458 binding, TM6a undergoes an inhibitor-induced transition and achieves a stable helical character with a mean helicity of 77.21% (Figure 3B; Figure S3), a 7-fold increase relative to that of the apo state (Figure 3A). This structural transition is even more evident in the superposition of the apo and bound states. Interestingly, the extent of TM6a formation in bound SPPL2a differs from that in bound PS1, where the latter contains fewer residues (Figure S4). A direct quantitative comparison of TM6a dynamics between SPPL2a and PS1 is not currently possible, as the equivalent characterization of TM6a in apo PS1 has not been performed, limiting its current structural evidence to inhibitor-bound snapshots only. However, since both SPPL2a and PS1 share the same catalytic architecture and inhibitor-induced TM6a appearance, it is plausible that PS1 undergoes a comparable disorder-to-order transition upon inhibitor binding. This makes the dynamic characterization of TM6a presented here for SPPL2a assume broader significance as a structural reference against which equivalent transitions in PS1 can be evaluated.

**Figure 3:**
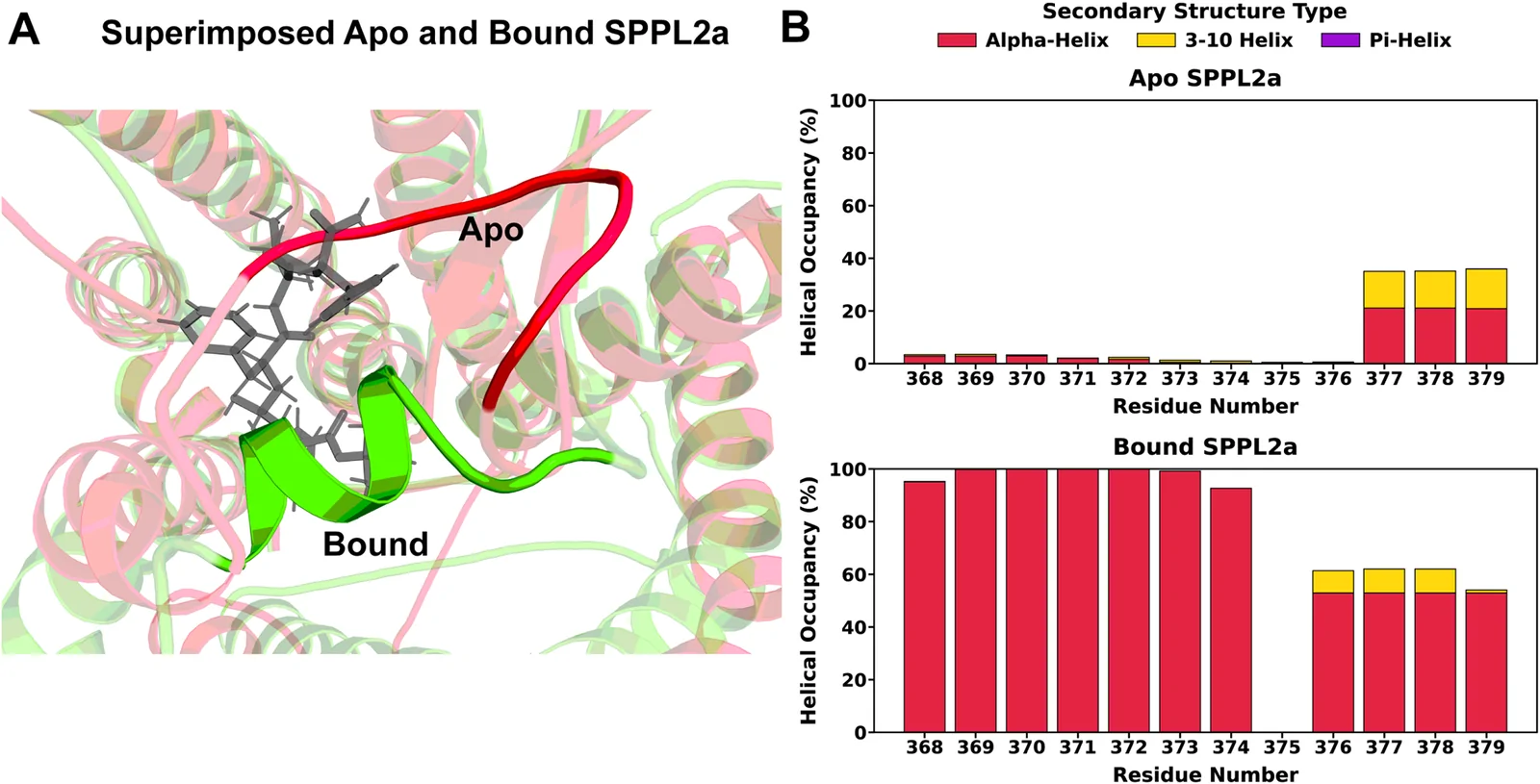
Transition of the TM6a region in apo and inhibitor-bound SPPL2a. (A) Structural superposition of apo SPPL2a (in pink) and L-685,458-bound SPPL2a (in green) focusing on the TM6a region. (B) Comparison of residuewise helical occupancy calculated across the complete trajectory for apo and inhibitor-bound SPPL2a.

### Global Conformational Dynamics and Interresidue Correlation Analysis of SPPL2a

To characterize the dominant motions of the proteins, PCA was performed on the backbone coordinates across the 1 μs trajectories, and the projection of the first two principal components (PC1 vs PC2) revealed a distinct conformational space sampled by the proteases (Figure 4A). Apo SPPL2a displayed the most expansive sampling of the three, with its PC1 capturing 51.3% of the total variance, reflecting a highly flexible ligand-free state where PC1 spanned a wide range from −99.8 to +146.1 Å. Visually, its conformation ensemble is broadly diffuse featuring a secondary populated region at high positive PC1 values that indicate the protease samples a wide landscape consistent with a highly flexible substrate-searching state. Cluster analysis resolved this into two states—an open configuration characterized by a lateral gate distance of 21.4 Å and catalytic dyad distance of 8.13 Å (Figure S5A), and a partially closed state with a contracted lateral gate of 17.5 Å and dyad distance of 7.78 Å, indicating that PC1 primarily describes lateral gate breathing between substrate accessible and intermediate conformations, consistent with PCA based segregation methods previously employed to resolve functionally discrete states in other membrane-associated proteins.^42^ Upon inhibitor binding, PC1 space was restricted between −72.0 to +77.6 Å and captured 31.8% of the variance, confirming that inhibitor binding globally restricts the conformational landscape of SPPL2a beyond the localized gate closure. This is resolved into two states: a rigid conformation with the lateral gate at its narrowest of 16.2 Å and dyad compressed to 7.27 Å (Figure S5B), and a comparatively relaxed state with dyad distance at 8.41 Å that nonetheless maintains the lateral gate tighter than any apo state configuration, indicating that the bound protease oscillates between two similar locked conformations. In comparison, the bound PS1 subunit exhibited the most constrained conformational phase space with PC1 ranging from −39.35 to 39.9 Å and capturing a variance of 29.4%. Cluster analysis resolved two closely related states with dyad distances of 3.1 and 3.4 Å (Figure S5C), indicating that compound E stabilizes an unusually compressed dyad geometry consistent with a catalytically suppressed conformation, reflecting minor localized oscillations and the stabilizing influence of the other γ-secretase subunits, whose interactions impose additional restraints on PS1 conformational freedom beyond inhibitor binding—a feature absent in the monomeric SPPL2a. To spatially resolve these global dynamics, the dominant motions along the first principal component were visualized using porcupine plots projected onto the architecture of the proteases, which illustrated the distinct directionality and magnitude of the collective fluctuations (Figure 4B–D).

**Figure 4:**
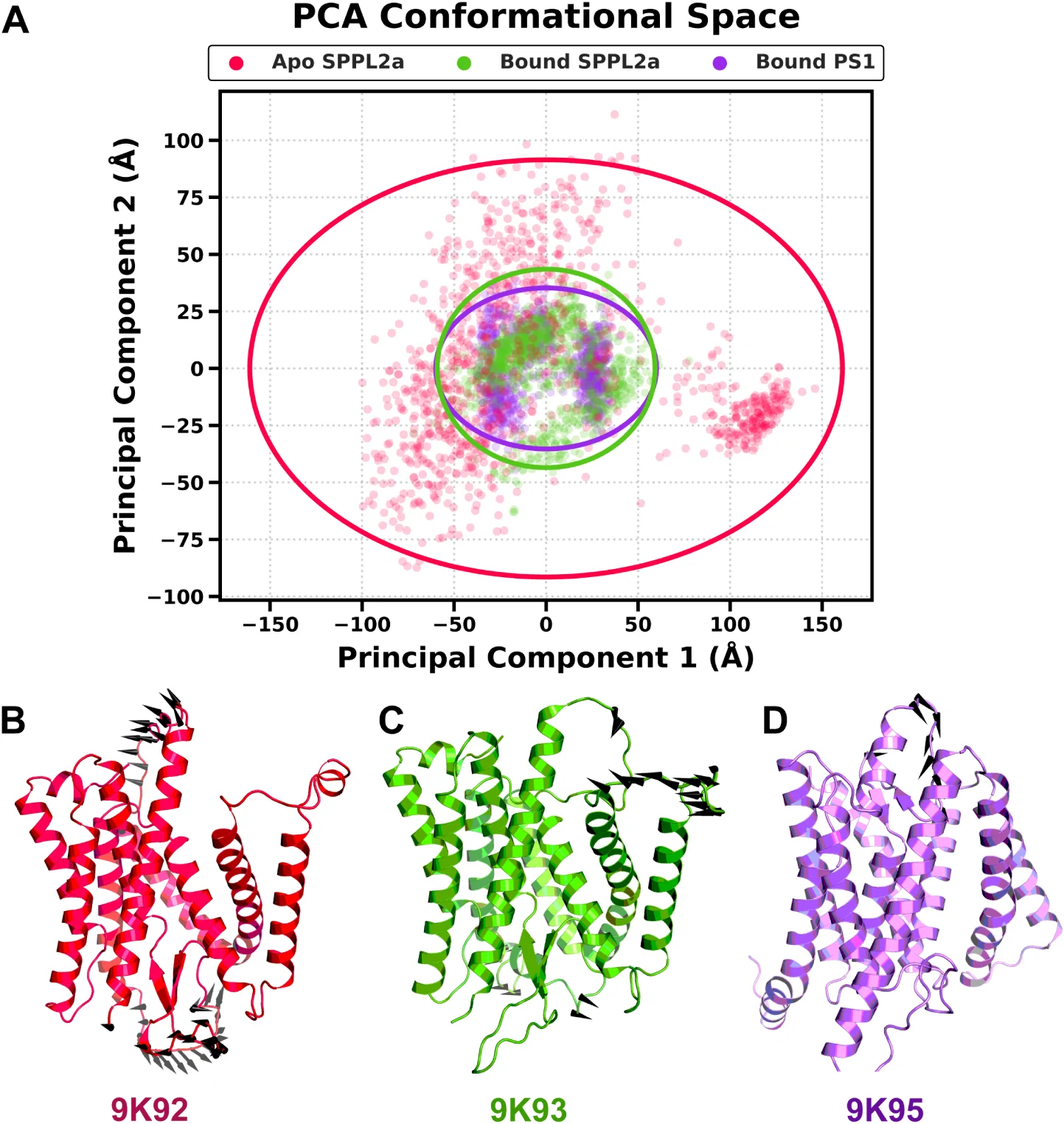
Principal component analysis and vector projection of SPPL2a and PS1 landscapes. (A) PCA scatter plot illustrating the conformational space explored in the simulation. The projection onto PC1 and PC2 highlights the clusters for the 3 systems. (B–D) Dominant collective motions of the simulated systems with the direction and magnitude of the first principal component (PC1) represented by black displacement vectors for (B) apo SPPL2a (C) inhibitor-bound SPPL2a (D) inhibitor-bound PS1.

While PCA maps the distinct conformational spaces traversed by the protein, it provides limited insight into the underlying residue-level communication. To understand how the protein coordinates these functions, DCCM was performed on the TM2, TM6, TM6a, and TM9 segments, quantifying the degree to which pairs of residues move in a correlated or anticorrelated motion across the trajectory (Figure S6). In apo SPPL2a, TM2 and TM6 moved in a slightly anticorrelated manner, reflecting their independent sampling as part of the open-gate ensemble, whereas TM2 and the disordered TM6a region maintained a modest positive correlation. TM2 and TM9 also moved in a correlated manner, indicating that the catalytic helix and the C-terminal bundle move as a loosely coordinated unit in the ligand-free state. However, inhibitor binding rewires this network: the previously anticorrelated TM2–TM6 interface shifts to a positive correlation. Moreover, the TM2–TM6a and TM6–TM9 correlations decreased, suggesting that while the gate and catalytic helix become locked together, their broader communication with TM6a and TM9 is broken. Overall, interresidue coupling decreases upon inhibitor binding, indicating that L685,458 selectively rewires its internal communication, reflecting a new dynamic unit consistent with the inhibitor-induced TM6a helix and the *de novo* contact network documented above.

### A Preformed Luminal Gate: Differential β-Hairpin Dynamics in SPPL2a and PS1

Among the structural features revealed by the cryo-EM studies of γ-secretase, perhaps none were more unexpected than the role of a short antiparallel β-hairpin, a seemingly unremarkable loop, in determining the precise position of substrates for intramembrane cleavage. In PS1, cryo-EM studies have established that substrate binding induces the formation of a hybrid β-sheet between the incoming substrate β-strand and the PS1 β-hairpin, with proteolytic cleavage occurring immediately after this contact.^15,43^ Like PS1, SPPL2a also has a β-hairpin; however, it is present in the apo state.^15^ To quantify β-hairpin stability, hydrogen bond occupancies were calculated across the trajectories, and in the apo state, the top contacts had occupancies of 93.4% and 92.0%, confirming that the hairpin is a constitutively stable structural element (Figure 5A). Upon inhibition, the top occupancy marginally decreased to 87.8%, suggesting that L685,458 induces partial conformational redistribution without loss of integrity (Figure 5B). In contrast to SPPL2a, where the β-hairpin is stable and marginally perturbed by inhibitor binding, the bound PS1 β-hairpin, unresolved in its apo cryo-EM structures, maintains stable contacts with a top occupancy of 88.5%, suggesting that inhibitor engagement may stabilize or induce hairpin formation in PS1 (Figure 5C). The same can be observed when the secondary structure stability is assessed (Figure 5D). This divergent inhibitor binding effect, where it stabilizes PS1 yet marginally destabilizes SPPL2a, is supported by the lack of sequence conservation observed in this β-hairpin region.^44^

**Figure 5:**
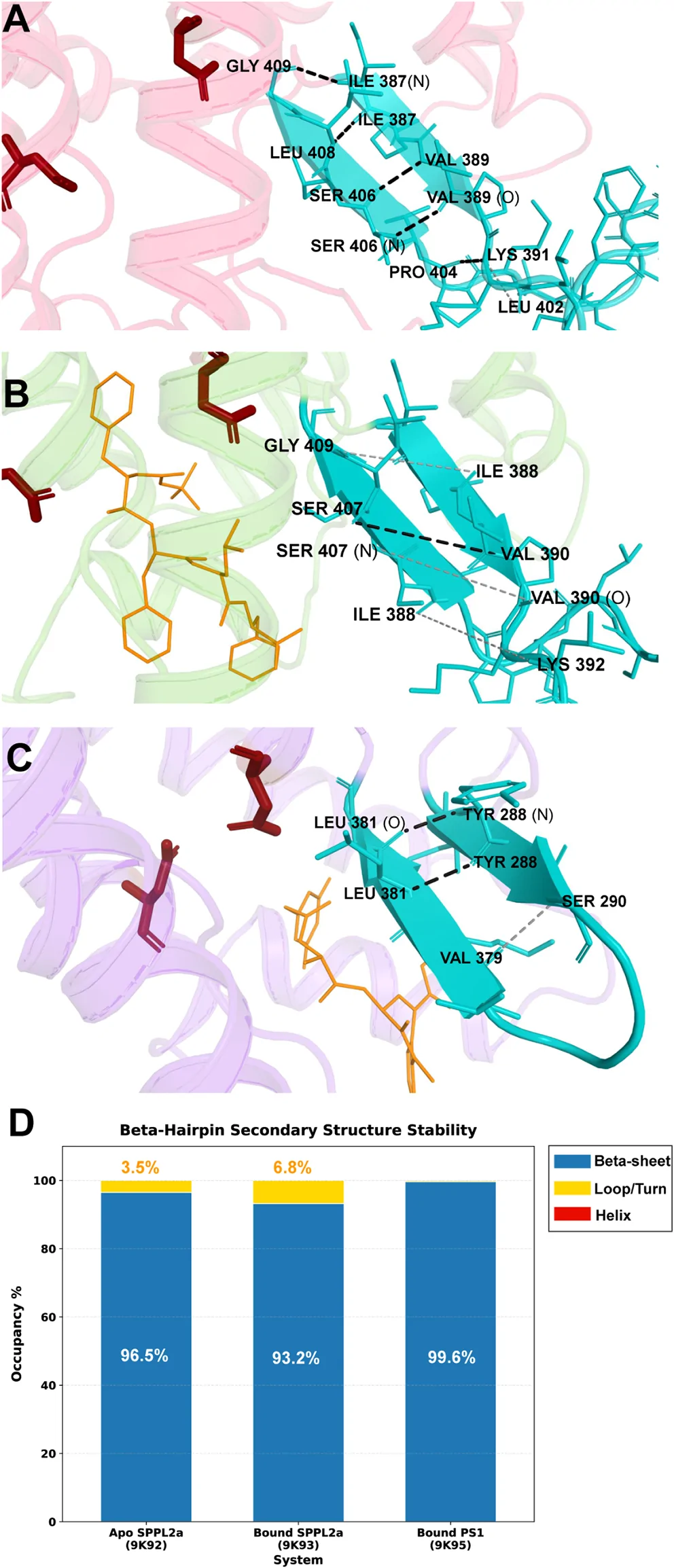
Comparative stability and hydrogen bond networks of the β-hairpin across proteases. (A–C) Inter-residue hydrogen bond occupancy within the β-hairpin of (A) apo-SPPL2a, (B) L-685,458-bound SPPL2a, and (C) inhibitor-bound PS1. The black dotted lines represent highly stable interactions with an occupancy ≥85%, whereas the gray dotted lines indicate moderate stability between 50% and 85% throughout the 1 μs trajectory. (D) Secondary structure stability across the three systems depicting the percentage of residues adopting specific secondary structural elements.

### Binding Free Energy Estimation and Energy Decomposition of SPPL2a and PS1

The binding free energy of the respective ligands of SPPL2a and PS1 within the active site was calculated using the MM-PBSA method for the last 200 ns (200 frames) of stable trajectories (Figure 6A). The total binding free energy (ΔG_bind_) for SPPL2a was −14.80 ± 3.29 kcal/mol, whereas it was −24.26 ± 3.14 kcal/mol for PS1 (Table 2), both of which may be slightly overestimated given the lack of entropic correction. However, it is also important to note that these values also reflect the distinct physicochemical properties of the two compounds as L-685,458 and Compound E differ substantially in molecular size, flexibility, and polar surface area. Consequently, the absolute ΔG_bind_ values across the two systems are not to be interpreted as a direct measure of comparative inhibitor potency or relative protease affinity, rather as a framework to characterize the inhibitor engagement in each system. With this noted, the decomposition of the energy into its constituent terms reveal that the van der Waals and electrostatic interaction energies were similar between SPPL2a and PS1, indicating that the direct noncovalent contacts at the inhibitor-binding interface are conserved across both proteases. Therefore, the thermodynamic distinction between the two systems is attributed to the solvation penalty—the desolvation cost upon inhibitor binding is considerably greater in SPPL2a than in PS1, a consequence of the differing sizes and polar surface areas of the two ligands rather than an inherent property of the binding pocket.

**Table 2:** Energetic Component Breakdown of Inhibitor Binding to SPPL2a and PS1 Calculated via the MM-PBSA Method

Energy component	Bound SPPL2a (9K93) (kcal/mol)	Bound PS1 (9K95) (kcal/mol)
ΔG_vdw_	–56.91 ± 4.02	–56.26 ± 3.1
ΔG_ele_	–9.15 ± 2.02	–9.06 ± 1.06
ΔE_pb_	13.96 ± 1.69	12.22 ± 0.97
ΔE_npolar_	–45.19 ± 2.47	–41.14 ± 1.64
ΔE_disper_	82.48 ± 3.64	69.99 ± 1.55
ΔG_gas_	–66.06 ± 4.1	–65.32 ± 3.24
ΔG_solv_	51.25 ± 3.11	41.07 ± 1.45
ΔG_total_	**–14.8 ± 3.29**	**–24.26 ± 3.14**

**Figure 6:**
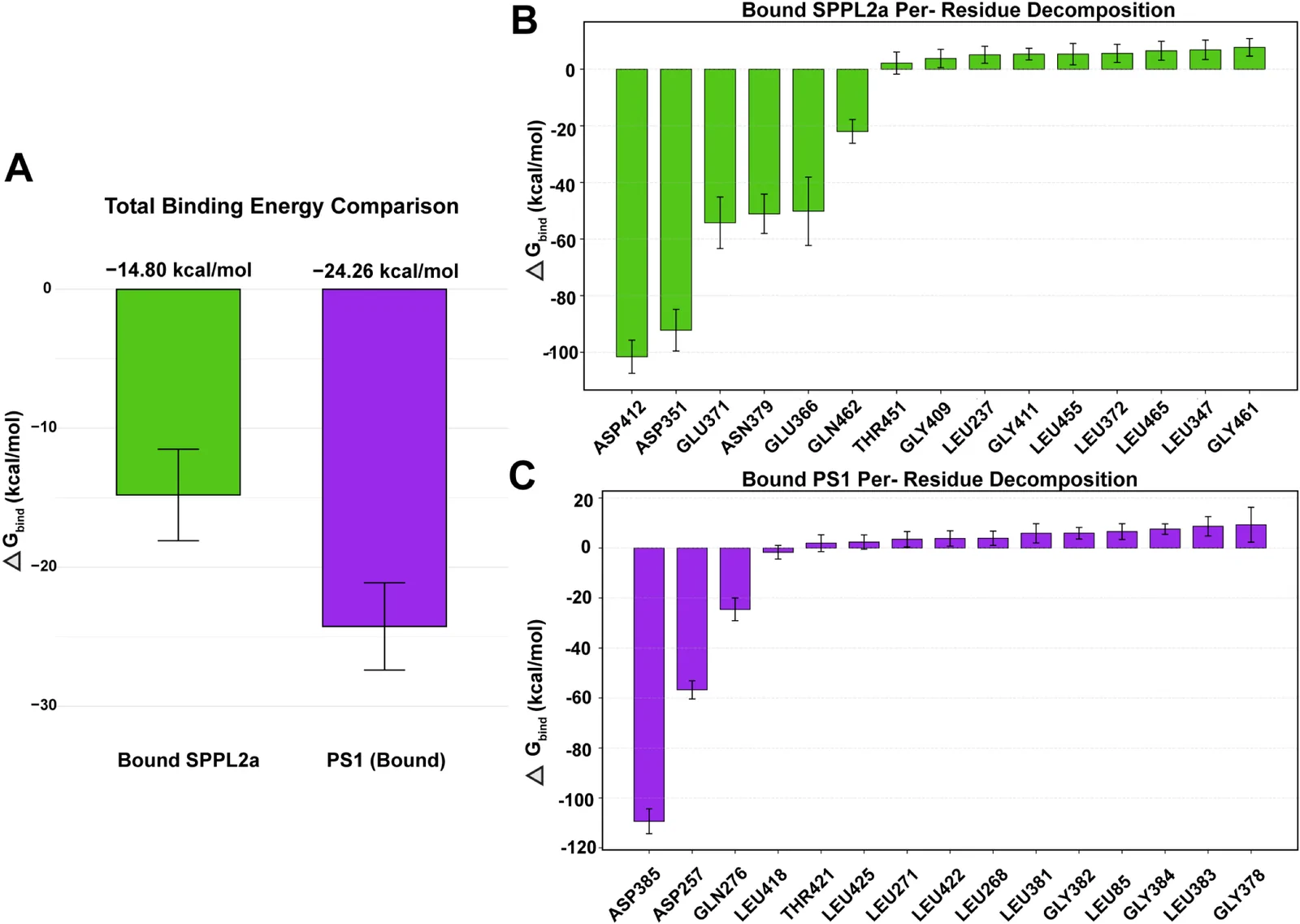
Binding free energy landscape and per-residue decomposition of inhibitor engagement. (A) Total binding free energy (ΔG_bind_) calculated using the MM-PBSA method for L-685,458 bound to SPPL2a and Compound-E bound to PS1. (B–C) Per-residue energy decomposition for (B) inhibitor-bound SPPL2a and (C) inhibitor-bound PS1, demonstrating the residues responsible for inhibitor binding.

Per-residue free energy decomposition revealed that catalytic aspartates were the dominant contributors to inhibitor binding in both systems (Table S1), which is consistent with the GxGD protease mechanism in which these residues coordinate the scissile bond of the substrate. In SPPL2a, the primary contributions were from a distributed polar network around the active site consisting of catalytic aspartates and the TM6a region (Figure 6B), whereas in PS1, the binding energy was more concentrated, with the catalytic aspartate 385 contributing much more than its equivalent in SPPL2a, as well as a minor hydrophobic contribution at the periphery of the binding interface (Figure 6C).

Taken together, the per-residue decomposition reveals that while the catalytic mode of recognition is conserved between SPPL2a and PS1, the binding energy of PS1 is concentrated at a single aspartate whereas SPPL2a relies on a broader set of polar residues—Glu 366, Glu 371 and Asn 379, compared to the hydrophobic or neutral equivalent region in PS1. This acts as a guide for drug design: first, the inhibitor scaffold can be modified to incorporate positively charged groups or hydrogen-bond donors to interact with Glu 366 and Glu 371, interactions that are structurally precluded in the more hydrophobic pocket of PS1. Second, engineering hydrophobic shielding groups adjacent to these basic groups of the inhibitor will displace water molecules and effectively shield Glu 366 and Glu 371 from the aqueous solvent, efficiently recovering the desolvation penalty to drive potency.

### Bilayer Structural Integrity and Dynamics of POPC Lipids

To assess whether protein embedding and inhibitor binding perturb the surrounding lipid bilayer, key membrane structural properties were characterized across all three systems throughout the 1 μs trajectories. Bilayer thickness, calculated as the center-of-mass distance between phosphorus atoms of opposing leaflets, remained stable at 39.20 ± 0.47 Å, 38.74 ± 0.47 Å, and 39.30 ± 0.44 Å for apo SPPL2a, bound SPPL2a, and the γ-secretase complex, respectively (Figure 7B), similar to the values observed in other studies.^45^ The marginal contraction upon L-685,458 binding suggests that SPPL2a reorganizes without providing mechanical stress to the surrounding bilayer. In γ-secretase, the slightly increased thickness of the upper leaflet compared with that of the lower leaflet is attributed to the influence of the large Nicastrin subunit, not an intrinsic property of PS1 and therefore not used as a basis of comparison. The APL values for both SPPL2a systems (∼59 Å^2^) are consistent with the expected packing for POPC bilayers in which a monomeric membrane protein is embedded, whereas the reduced APL observed for γ-secretase (53.53 ± 14.82 Å^2^) reflects the greater constraints imposed by the larger architecture on the surrounding lipid mobility (Figure 7D; Figure S7).

**Figure 7:**
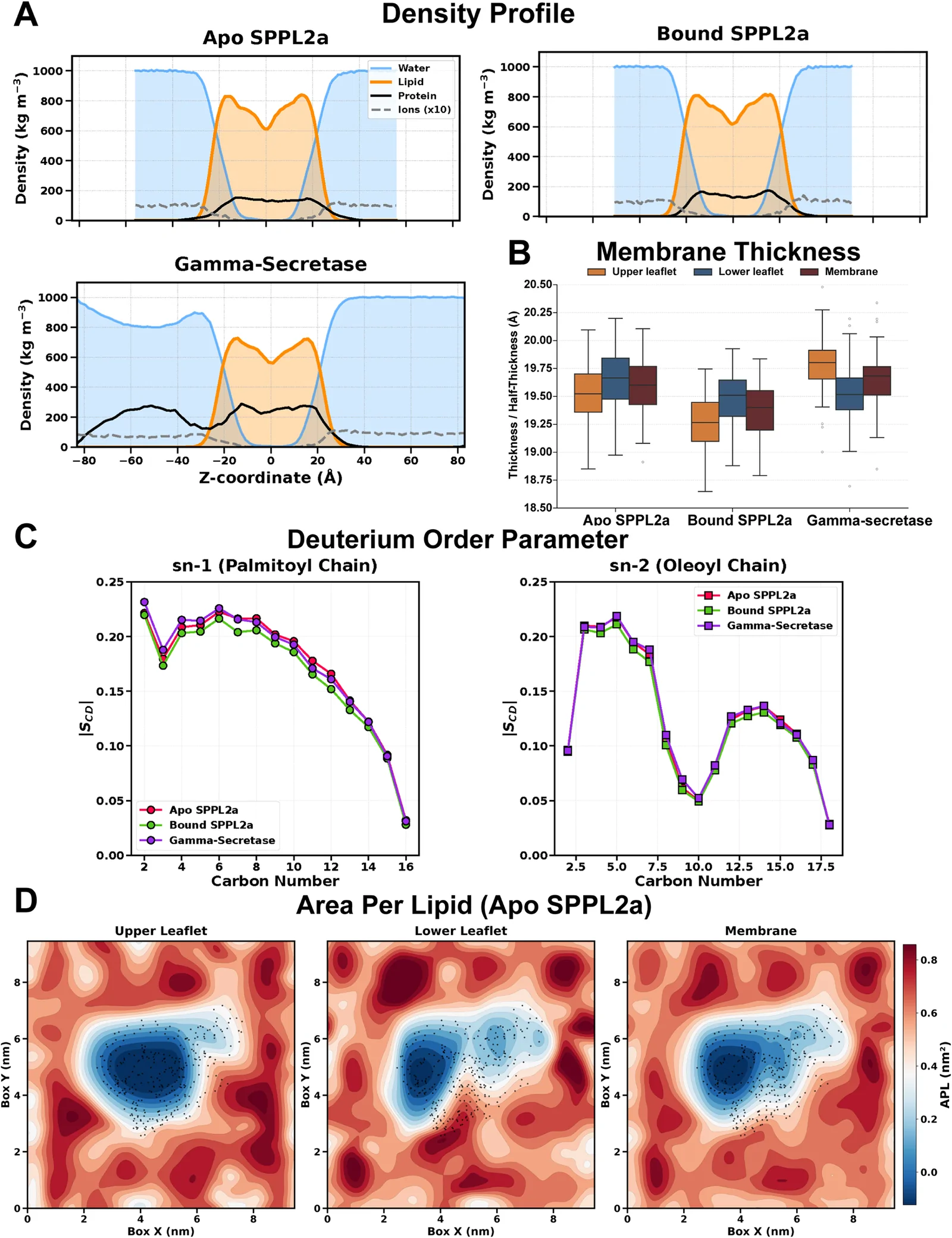
Assessment of lipid bilayer properties and structural integrity. (A) Partial density distributions of membrane components calculated along the bilayer normal. (B) Boxplots representing the average bilayer thickness for the upper leaflet, lower leaflet, and total membrane across all the systems. (C) Lipid acyl chain order parameters. (D) Contour plots demonstrating the local lipid organization around the protein–transmembrane interface of apo SPPL2a.

Beyond these, the deuterium order parameters (SCDs) were calculated and found to be consistent across all the simulation systems, with sn-1 values of 0.1736, 0.1660, and 0.1741 and sn-2 values of 0.1266, 0.1225, and 0.1276 for apo, bound SPPL2a, and γ-secretase, respectively (Figure 7C). The density distribution profiles along the bilayer normal demonstrate a stable membrane component distribution with the water density decreasing to zero between Z = −20 and +20 Å across all three systems, providing evidence of a well-sealed, physically realistic membrane environment maintained throughout the microsecond time scale (Figure 7A).

PyLipID analysis, which quantified lipid interaction kinetics across the 1 μs trajectories, revealed that POPC contacts with the transmembrane domains of SPPL2a and PS1 extend beyond nonspecific annular shell interactions, identifying discrete residue-level binding hotspots. In apo SPPL2a, the lipid–protein interface was characterized by a broadly distributed contact network—149 residues had an occupancy > 50% and duration (residence time) > 50 ns, with relatively balanced residence times across the TM helices, suggesting a fluid interface. The prominent contacts were Leu 192 (360 ns), Phe 452 (270 ns), Cys 263 (260 ns), and Tyr 437 (258 ns), residues distributed across the TMs rather than concentrated, indicating a nonspecific lipid engagement pattern in the ligand-free state (Figure 8A).

**Figure 8:**
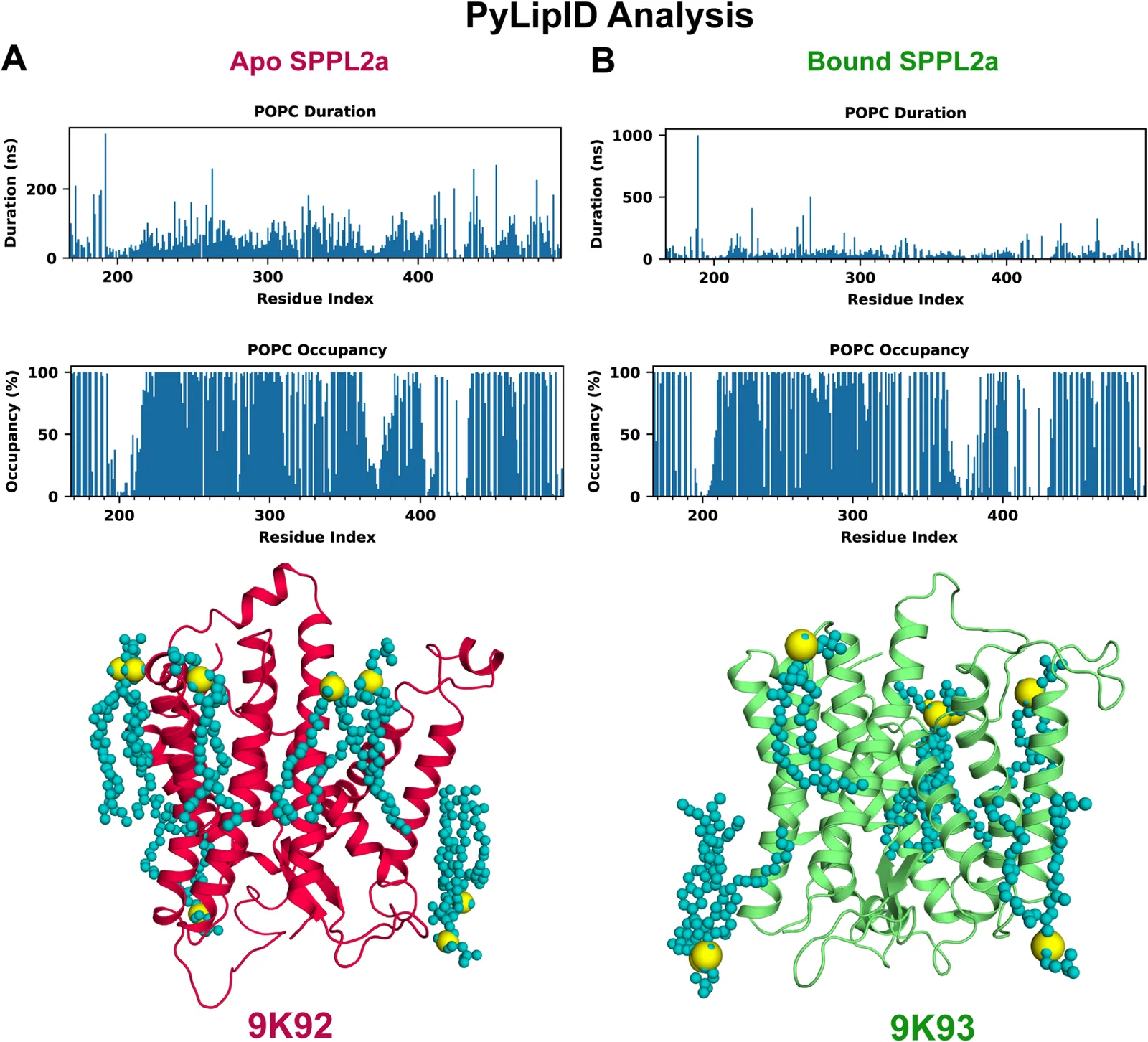
Analysis of site-specific lipid–protein interactions in the apo and bound states of the aspartyl protease SPPL2a using PyLipID. Duration (residence time) and occupancy percentage calculated for POPC lipids, along with structural representations illustrating the persistent binding locations across (A) apo SPPL2a and (B) bound SPPL2a.

A wave of changes appears upon inhibitor binding—while the contact network is reduced to 127, the topmost contact—Trp 189—maintains a locked lipid contact throughout the 1 μs simulation. Other notable contacts include Gln 462 (326 ns), a residue previously identified as forming critical contacts with L-685,458,^15^ suggesting that inhibitor-induced conformational change extends to the lipid environment, creating a newly stabilized protein–lipid interface as a consequence of intramolecular reorganization and a shift from distributed interactions toward localized, site-specific hotspots (Figure 8B). On analysis of the PS1 subunit from the γ-secretase complex, we found 132 residues whose occupancy exceeded 50% and whose duration was >50 ns. Among these, Pro 218 serves as a structural anchor with 100% occupancy and rigid contact throughout the 1 μs simulation, and additional key residues, including Gln 223 and Tyr 240, are present at the ends of the TMs, suggesting their role in preserving the stability of the protease (Figure S8).

Establishing these lipid-contact profiles is a significant first step toward resolving the broader regulatory logic of SPPL2a. Given that lipid modulation is a well-established hallmark of γ-secretase activity,^46,47^ our results suggest that SPPL2a is likely subject to similar lipid-mediated control, providing an essential starting point for future functional investigations.

The bilayer parameters reported here raise a question: does the structural behavior of the membrane correlate with SPPL2a catalytic competence? Evidence from related intramembrane proteases indicate that the surrounding lipid environment actively regulates catalytic function rather than being a passive scaffold. For instance, reconstitution of γ-secretase into defined lipid mixtures has shown that bilayer composition modulates both rate and processivity of substrate cleavage.^48,49^ Similarly, the activity of rhomboid protease GlpG correlates with bilayer hydrophobic thickness, peaking within a defined thickness window only due to a pattern explained by a hydrophobic mismatch between the transmembrane domain and surrounding lipids.^50,51^ These examples demonstrate that bilayer physical dimensions and fluidity constrain the conformational changes required for substrate engagement and processing across serine and aspartyl intramembrane hydrolases.

As a GxGD-type aspartyl protease, SPPL2a is expected to be subject to identical physical constraints. The bilayer thickness values obtained here for apo and inhibitor-bound SPPL2a fall within the range associated with permissive catalytic geometry in these reference systems, and the modest contraction observed upon L-685,458 binding follows the same direction as the thinning linked to enhanced transmembrane accessibility in GlpG,^51^ placing the membrane environment sampled in our simulations within a structurally favorable regime for SPPL2a function.

### Protonation State Sensitivity Analysis of Apo SPPL2a

To evaluate whether the doubly deprotonated assignment of the catalytic dyad influences the results obtained from the apo trajectory, an additional 1 μs simulation of apo SPPL2a with Asp 351 protonated (by homology to PS1 Asp 257)^52^ was performed and compared against the original system across key structural metrics (Figure S9). Global structural integrity was preserved as evident from the RMSD value of 0.48 ± 0.03 nm on convergence compared to 0.52 ± 0.02 nm for the deprotonated system, and an identical R_g_ value, as well as comparable β-hairpin stability. However, protonation compressed the catalytic dyad Oδ−Oδ distance to 4.79 Å from 7.78 Å consistent with stabilization of an interdyad hydrogen bond, and suppressed TM6a helicity from 10.36% to 2.44%, indicating that dyad protonation state modulates TM6a folding propensity in the ligand-free state. Critically, these indicate that the principal findings of this study are derived from structural regions and time scales insensitive to this active-site perturbation, and the doubly deprotonated apo system therefore provides a sufficient baseline for the conformational conclusions drawn here.

While this work provides a detailed framework of SPPL2a dynamics, recognizing the limitations of this research clarifies both its immediate contributions and the prospective frontiers it enables. We utilized a homogeneous POPC bilayer to ensure a stable environment for our trajectories, following established computational standards for intramembrane proteases.^53^ However, we acknowledge that the lysosomal membrane where SPPL2a resides is enriched with lipids such as BMP, cholesterol, and sphingomyelin, which are components known to modulate the conformational landscapes of membrane proteases.^46^ Transitioning to these more complex, physiologically realistic lipid mixtures represents a next step in refining the structural behavior of the enzyme. Next, although the bilayer geometry sampled here is consistent with a catalytically permissive geometry by analogy to γ-secretase and GlpG, this remains a structural inference only, and establishing a direct causal relationship with SPPL2a would require systematically varying the lipid bilayer composition across a panel of MD simulations and parallel *in vitro* biochemical assays in the presence of physiologically relevant substrates. Furthermore, this study focused specifically on the mechanism of inhibition rather than the process of substrate catalysis and was designed on the basis of a static snapshot study.^15^ Future simulation efforts focusing on substrate capture, which will require protonation of one of the catalytic aspartates, will add to the foundational understanding of the inhibited ensemble. Also, interpretation of these simulations require accounting for the structural asymmetry between the multisubunit γ-secretase complex (PS1, Nicastrin, APH-1 and PEN-2) and the monomeric SPPL2a. The system-level metrics such as RMSD, radius of gyration, and PCA-derived variance reflect the conformational behavior of PS1 within the constraints of its native multisubunit assembly rather than its intrinsic flexibility alone. Consequently, the comparisons drawn between SPPL2a and PS1 in this study are weighted toward features that are local to the catalytic core that are less sensitive to this compositional asymmetry, namely the catalytic aspartate network, β-hairpin hydrogen bonding, and per-residue energetic contributions, whereas the system-wide metrics are presented purely to provide descriptive architectural context for each protease. This distinction does not diminish the value of contrasting the two proteases, which remain the most structurally informative pairing available for SPPL2a, but it defines the scope within which such comparisons should be interpreted. Ultimately, each of these areas represent not flaws but frontiers, defining an extension of the work. This study provides the essential starting point, establishing the dynamic vocabulary necessary to pursue the selective inhibition of a protease whose therapeutic relevance, AD, is only beginning to be appreciated.

## Conclusion

The present study reports the first microsecond-time-scale, all-atom molecular dynamics characterization of human SPPL2a in its apo and inhibitor-bound states, alongside a parallel analysis of the PS1 catalytic subunit of γ-secretase, which collectively provides an atomic-resolution dynamic framework for the GxGD intramembrane aspartyl protease family member that has, until now, existed only as static cryo-EM snapshots.^15^ The therapeutic urgency of this work is illuminated by two key developments—the failure of γ-secretase inhibitors in clinical trials due to their massive side effects^6^ and the growing genetic and functional evidence implicating SPPL2a in AD pathogenesis because of its processing of the substrate TMEM106B and its association with AD risk loci in GWAS.^10^ With this background in mind, an atomistic understanding of what distinguishes SPPL2a from PS1 is not just a matter of mechanistic interest but also a prerequisite for selectivity-guided drug design.

The microsecond simulations presented here establish several mechanistically significant findings that were previously inaccessible. Our simulations reveal that SPPL2a is a conformationally dynamic enzyme whose ligand-free state is defined by broad conformational sampling and a sparse interhelical contact network whose architecture is completely dismantled upon inhibitor engagement. TM6a, resolved in apo SPPL2a but not in apo PS1 cryo-EM structures, is here quantitatively characterized for the first time in any GxGD protease: disordered at 10.36% helicity in the apo state, it folds to 77.21% upon inhibitor binding, thus establishing the persistence of this transition at microsecond resolution, a benchmark against which equivalent transitions in PS1 can now be evaluated. The dynamic communication network is also fundamentally rewired—TM2 and TM6 shift from anticorrelated to positively correlated motion, while TM2 decouples from TM6a, collectively redefining L685,458 not as an active site plug but as an allosteric conformational refurbisher of the entire TM bundle. Finally, an inhibitor-stabilized lipid hotspot at Trp189, which is absent in the apo state and persists throughout the full 1 μs trajectory, reveals that inhibitor-induced conformational change also propagates certain TMs outward to the lipid-facing surface, a regulatory dimension of SPPL2a that could not have been identified by cryo-EM alone.

Crucially, several features characterized here offer avenues for selectivity-guided drug design against SPPL2a over PS1. The distributed binding energy architecture of SPPL2a, which is spread across a polar network around the active site rather than concentrated at a single aspartate, as in PS1, suggests that extended pharmacophores bridging the catalytic dyad and the TM6a-forming region may achieve selectivity that has eluded γ-secretase-targeted trials. The inhibitor-stabilized lipid hotspot at Trp189, absent in the PS1 lipid profiles, represents a potential allosteric surface for the membrane-anchored approach. Additionally, the distinct β-hairpin dynamics, where inhibitor binding modestly destabilizes the preformed gate in SPPL2a while coinciding with the emergence of stable contacts in PS1, identify the hairpin as a further structural locus whose differential behavior could be exploited for selectivity. These hypotheses are grounded directly in dynamic data unavailable prior to this study.^44^ Ultimately, these simulations resolve the conformational landscape of SPPL2a transitioning it into a therapeutic target to guide selective drug discovery.

## Supplementary Material



## Data Availability

The molecular dynamics trajectory files (.gro, .xtc, and .tpr) for all the simulated systems, along with their replicates, are publicly available on Zenodo at 10.5281/zenodo.20195564. The Python and shell scripts used for the global metrics, protein and lipid analyses, and data visualization are hosted on GitHub at https://github.com/sagnicode-dutta/SPPL2a-Dynamics.

## Notes

Additional figures and tables accompanying this manuscript: triplicate convergence and reproducibility plots for all three MD simulation systems; RMSD and secondary structure-based global convergence profiles for SPPL2a and γ-secretase trajectories; time evolution of TM6a secondary structure propensity across the simulation; structural superposition of L-685,458-bound SPPL2a and PS1 highlighting the TM6a region; distance profiles of the catalytic dyad across conformational clusters; comparative dynamic cross-correlation maps (DCCM) for apo and inhibitor-bound SPPL2a; area-per-lipid plots for all three simulated systems; PyLipID-derived site-specific lipid–protein interaction profiles for the PS1 subunit of the γ-secretase complex; comparative apo SPPL2a protonation-state dynamics; and per-residue binding free energy decomposition values for inhibitor interactions with SPPL2a and PS1 (PDF)
