# Supplementary material for: Unlocking the Dynamics of Human SPPL2a: First Atomistic Characterization of an Alzheimer’s-Linked Intramembrane Protease
Source: J Chem Inf Model. 2026 Jul 20;66(16):10088–102. doi: 10.1021/acs.jcim.6c01556 (PMC13508776; doi:10.1021/acs.jcim.6c01556)
Supplement: Supplementary file 1 [file ci6c01556_si_001.pdf]

Supporting Information

Unlocking the Dynamics of Human SPPL2a: First Atomistic Characterization of an Alzheimer's-Linked Intramembrane Protease

Sagnika Dutta[1], Mahender Kumar Singh[2], Budheswar Dehury[1]*

[1]Department of Bioinformatics, Manipal School of Life Sciences, Manipal Academy of Higher Education, Manipal-576104, India

[2]Data Science Laboratory, National Brain Research Centre, Gurgaon, Haryana, 122052, India

***Corresponding Author**

Dr. Budheswar Dehury

Email: budheswar.dehury@manipal.edu

List of Table and Figures:

Table/ Figures	***Content***	***Page No.***
Table S1	Per-residue binding free energy decomposition of inhibitor interactions with SPPL2a and PS1.	S3
Figure S1	Convergence and reproducibility of MD simulations across triplicate runs for all three systems.	S4
Figure S2	Structural stability and global convergence of SPPL2a and γ-secretase MD trajectories.	S5
Figure S3	Evolution of the secondary structure propensity within the TM6a region across the complete trajectory.	S6
Figure S4	Superposition of PS1 and L-685,458 bound SPPL2a highlighting the TM6a region.	S7
Figure S5	Distance profiles of the catalytic dyad across distinct conformational clusters.	S8
Figure S6	Comparative dynamic cross-correlation maps (DCCM) in intramembrane protease SPPL2a.	S9
Figure S7	Area per Lipid plots of the intramembrane aspartyl protease systems.	S10
Figure S8	PyLipID analysis of site-specific lipid-protein interactions in bound PS1 subunit of the γ-secretase complex.	S11
Figure S9	Comparative structural and conformational dynamics of apo SPPL2a across protonation states.	S12

Table S1. Per-residue binding free energy decomposition of inhibitor interactions with SPPL2a and PS1

System	Residue	vdW (*kcal/mol*)	Electrostatic (*kcal/mol*)	Polar Solvation (*kcal/mol*)	Non-Polar Solvation	Total Contribution (*kcal/mol*)
**Bound SPPL2a (9K93)**	Asp:351	-3.38	-78.71	1.5	0	-38.06
	Glu:371	-5.12	-103.57	-3.81	0	-36.06
	Asn:379	-3.75	-44.85	-0.62	0	-34.04
	Glu:366	-2.58	-100.31	-4.79	0	-33.46
	Gln:462	-3.81	-36.89	-0.39	0	-9.76
	Thr:451	-4.75	-37.97	-0.19	0	1.53
	Gly:409	-3.15	-49.83	0.17	0	2.69
	Leu:455	-4.25	-26.9	-0.09	0	3.01
	Leu:237	-6.33	-32.46	-0.06	0	3.4
	Gly:461	-1.45	-29.79	-0.18	0	3.43
**Bound PS1 (9K95)**	Asp:257	-8.24	-69.3	-0.4	0	-56.74
	Gln:276	-3.64	-57.53	-1.77	0	-16.1
	Leu:418	-6.32	-32.05	0.2	0	-1.07
	Leu:425	-4.55	-26.07	0.05	0	1.33
	Thr:421	-4.72	-37.71	-0.17	0	1.39
	Leu:271	-6.59	-34.13	0	0	2.51
	Gly:382	-2.22	-34.43	-0.11	0	3
	Leu:381	-3.17	-31.37	0.15	0	3.41
	Leu:268	-5	-34.58	0.08	0	3.63
	Leu:422	-5.52	-42.95	0.04	0	4.22

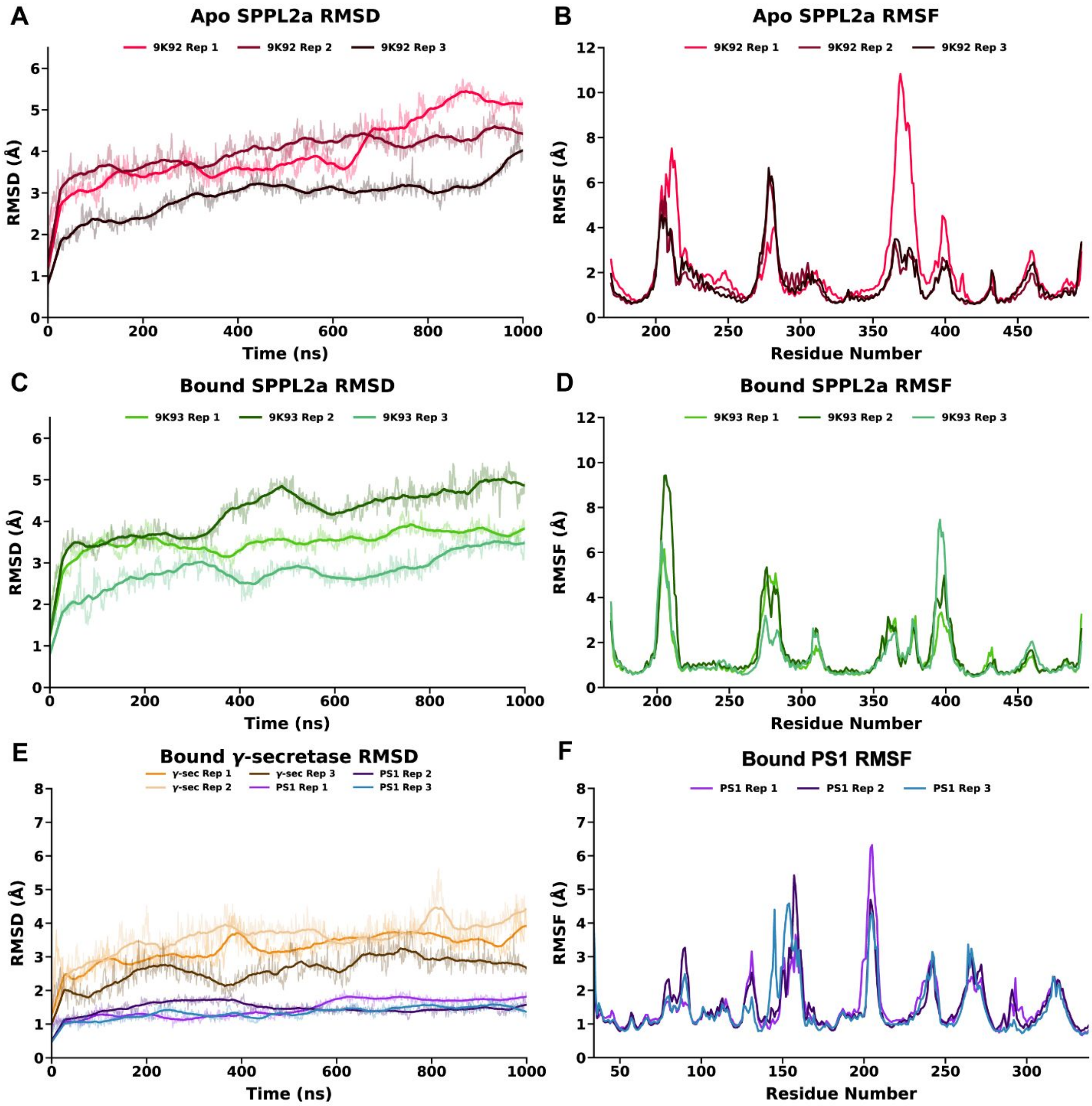

Figure S1. Convergence and reproducibility of MD simulations across triplicate runs for all three systems. (A) Backbone RMSD of apo SPPL2a plotted over the full trajectory for three independent replicates. **(B)** RMSF of apo SPPL2a across three independent replicates. **(C)** Backbone RMSD of L-685,458 bound SPPL2a over the 1 µs trajectory for three independent replicates. **(D)** RMSF of inhibitor-bound SPPL2a across three independent replicates. **(E)** Backbone RMSD of the compound E-bound γ-secretase and its extracted subunit PS1 over the full trajectory. **(F)** RMSF of the Compound-E bound PS1 across three independent replicates. In all panels, replicates are distinguished by color (Rep 1, Rep 2, Rep 3).

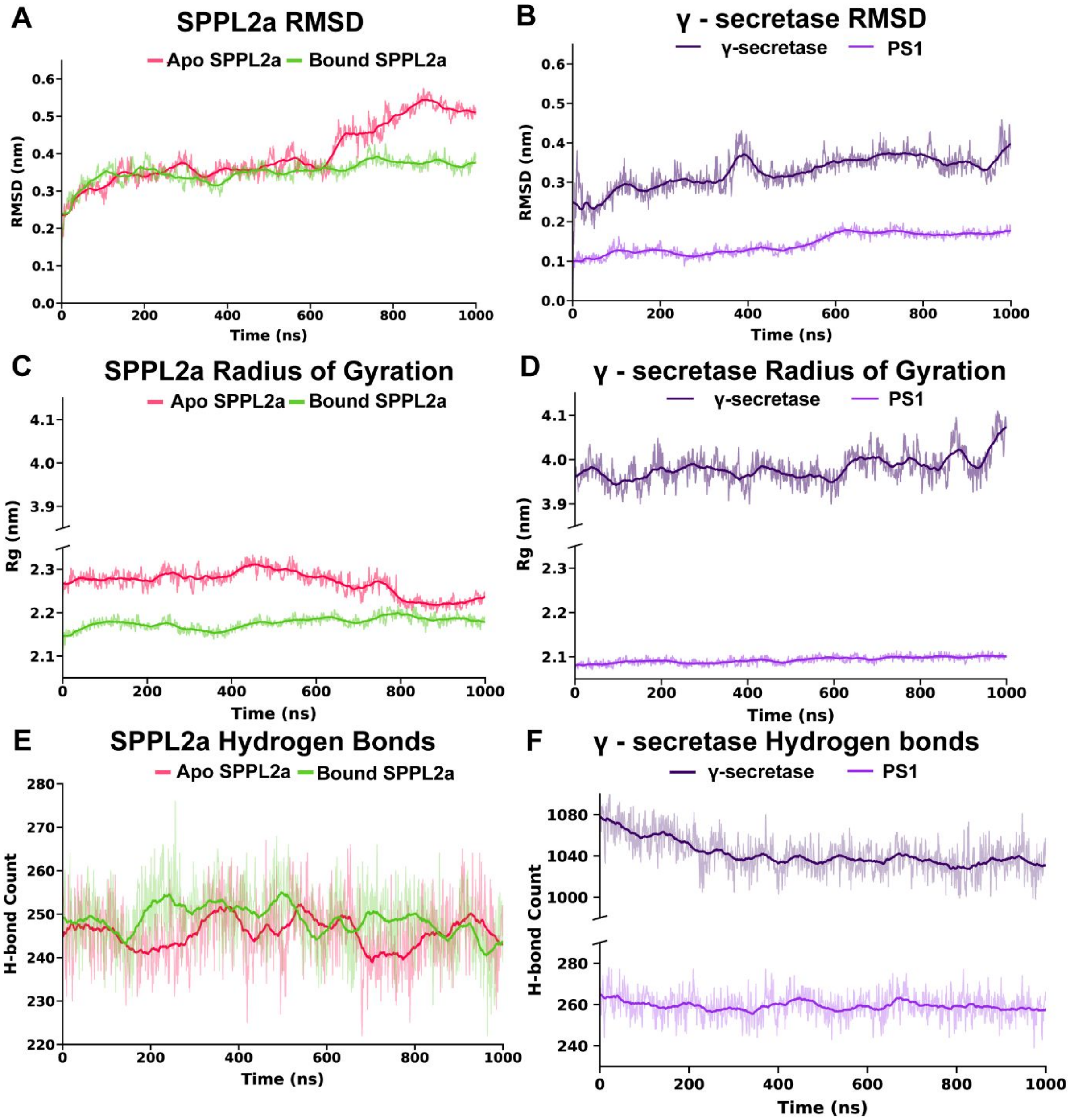

Figure S2. Structural stability and global convergence of SPPL2a and γ-secretase MD trajectories. (A) Root Mean Square Deviation of the protein backbone for apo (pink) and bound (green) SPPL2a over a 1 µs trajectory. **(B)** Root Mean Square Deviation of the protein backbone for γ-secretase (deep violet) and its extracted subunit PS1 (purple) over a 1 µs trajectory. **(C)** Radius of gyration analysis illustrating the global compactness of apo and bound SPPL2a. **(D)** Radius of gyration analysis illustrating the global compactness of γ-secretase and its extracted subunit PS1. **(E-F)** Time-evolution of total internal hydrogen bonds within the protein systems SPPL2a and γ-secretase.

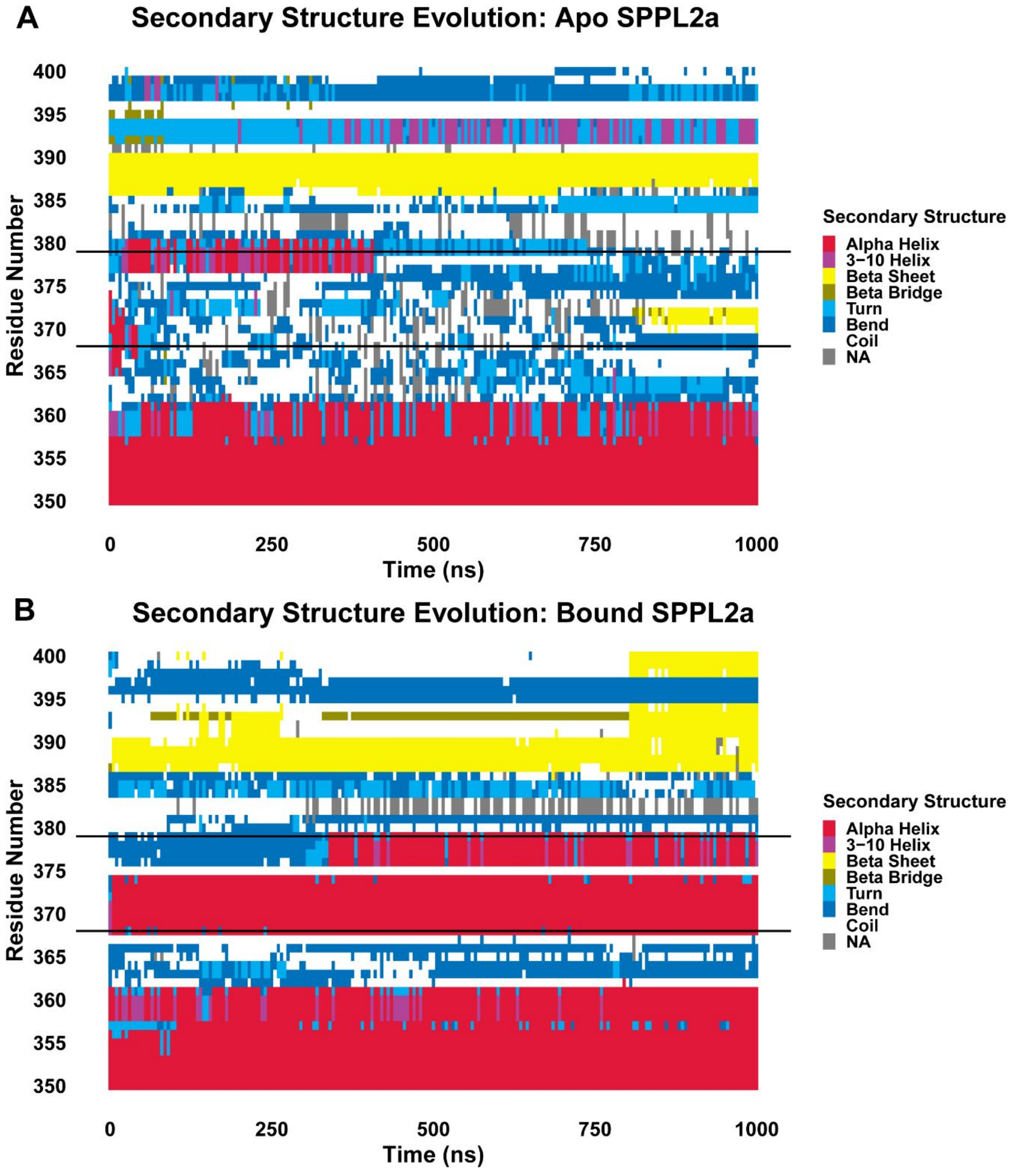

Figure S3. Evolution of the secondary structure propensity within the TM6a region across the complete trajectory. DSSP profiles for the TM6a segment in **(A)** apo SPPL2a and **(B)** L-685,458 bound SPPL2a. The black horizontal lines mark the TM6a segment regions in the panel.

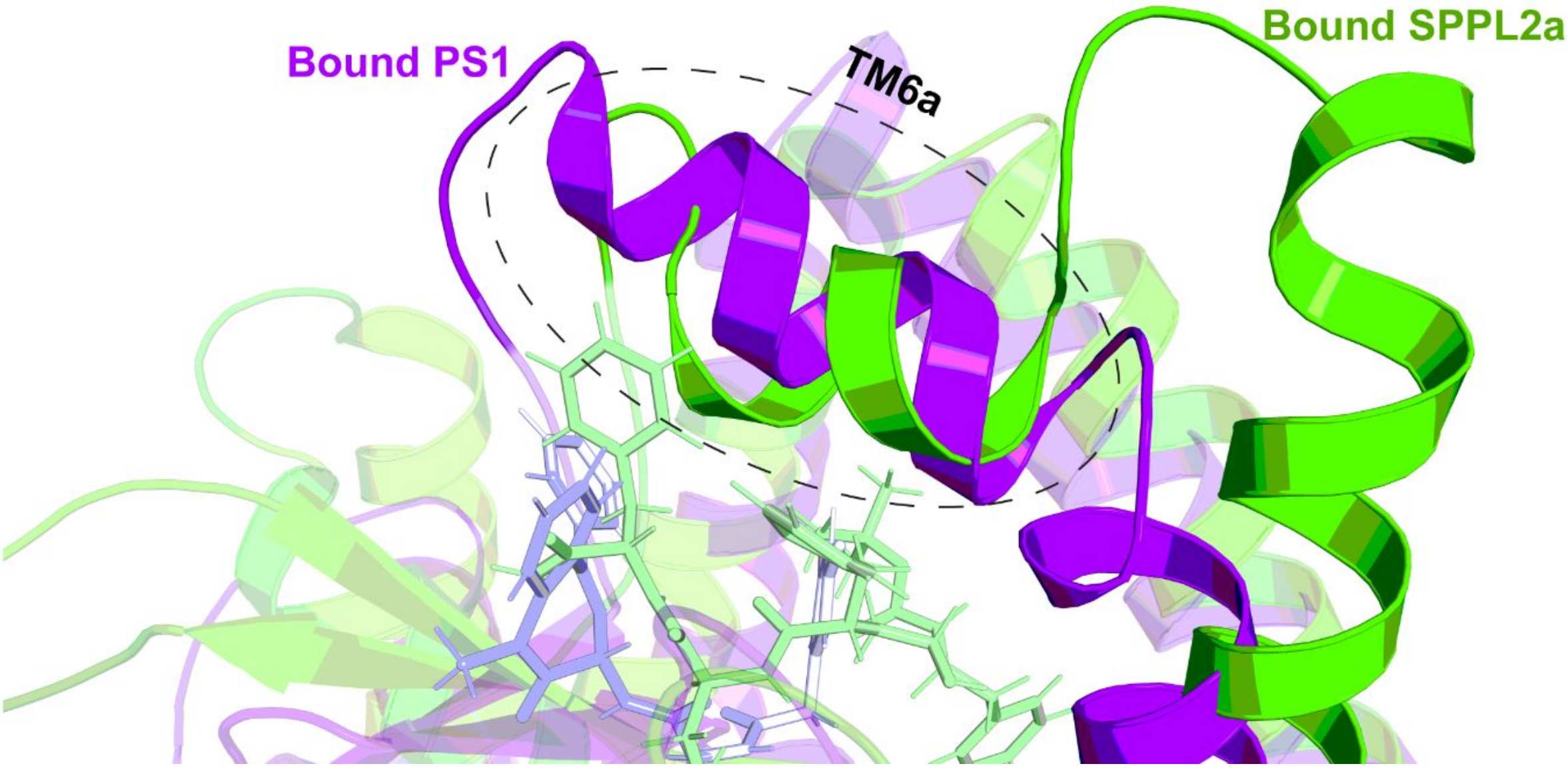

Figure S4. Superposition of PS1 and L-685,458 bound SPPL2a highlighting the TM6a region. The image demonstrates how the TM6a region for PS1 (in purple) contains more residues in helix than SPPL2a (in green).

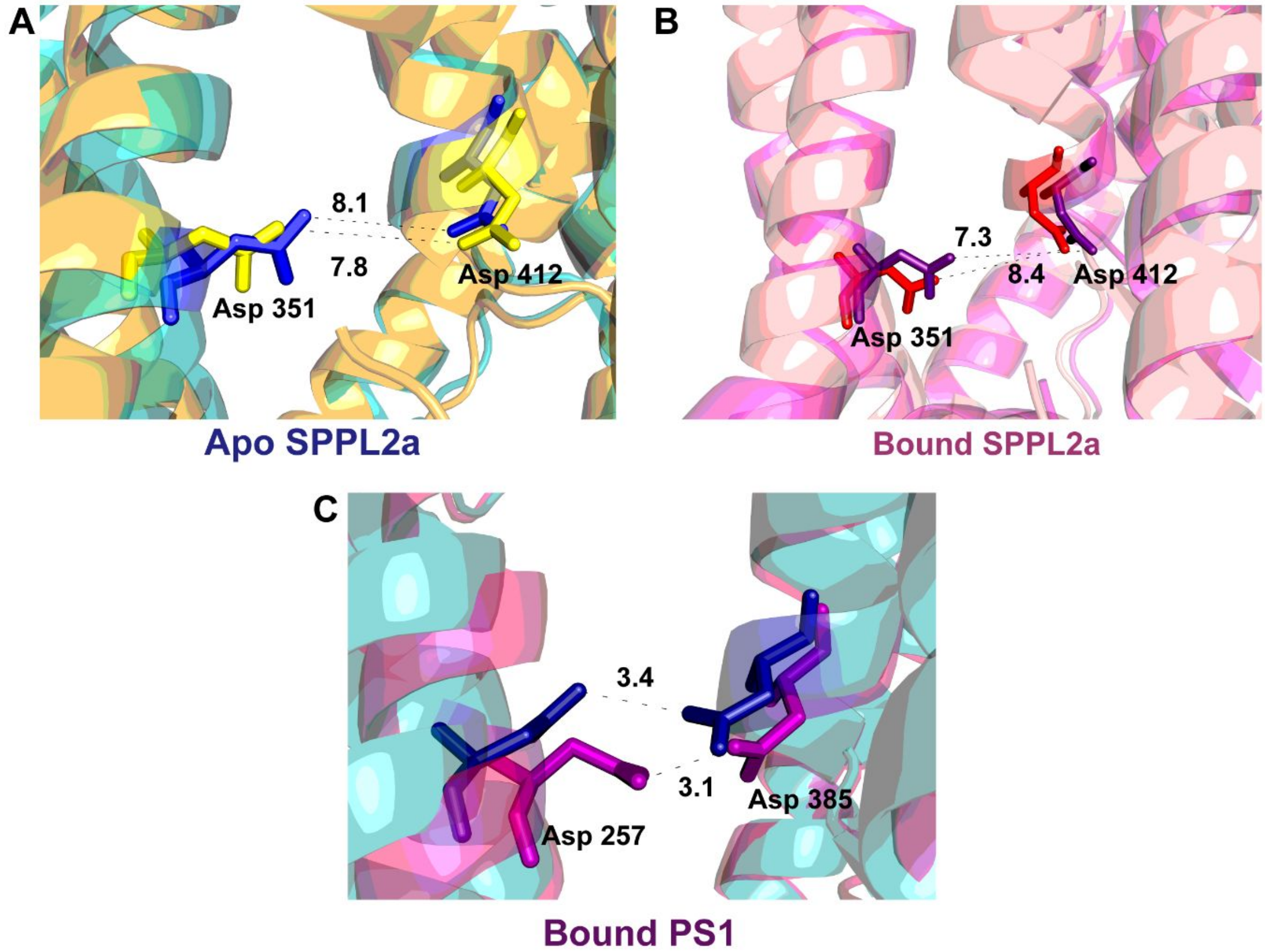

Figure S5. Distance profiles of the catalytic dyad across distinct conformational clusters. PyMOL structural representations highlighting Oδ–Oδ distances between the catalytic aspartate residues of **(A)** Apo SPPL2a with Asp 351 and Asp 412 highlighted as blue and yellow sticks in cluster one and two. **(B)** Bound SPPL2a with Asp 351 and Asp 412 highlighted as red and violet sticks in cluster one and two. **(C)** Bound PS1 with Asp 257 and Asp 351 highlighted as blue and magenta sticks in cluster one and two.

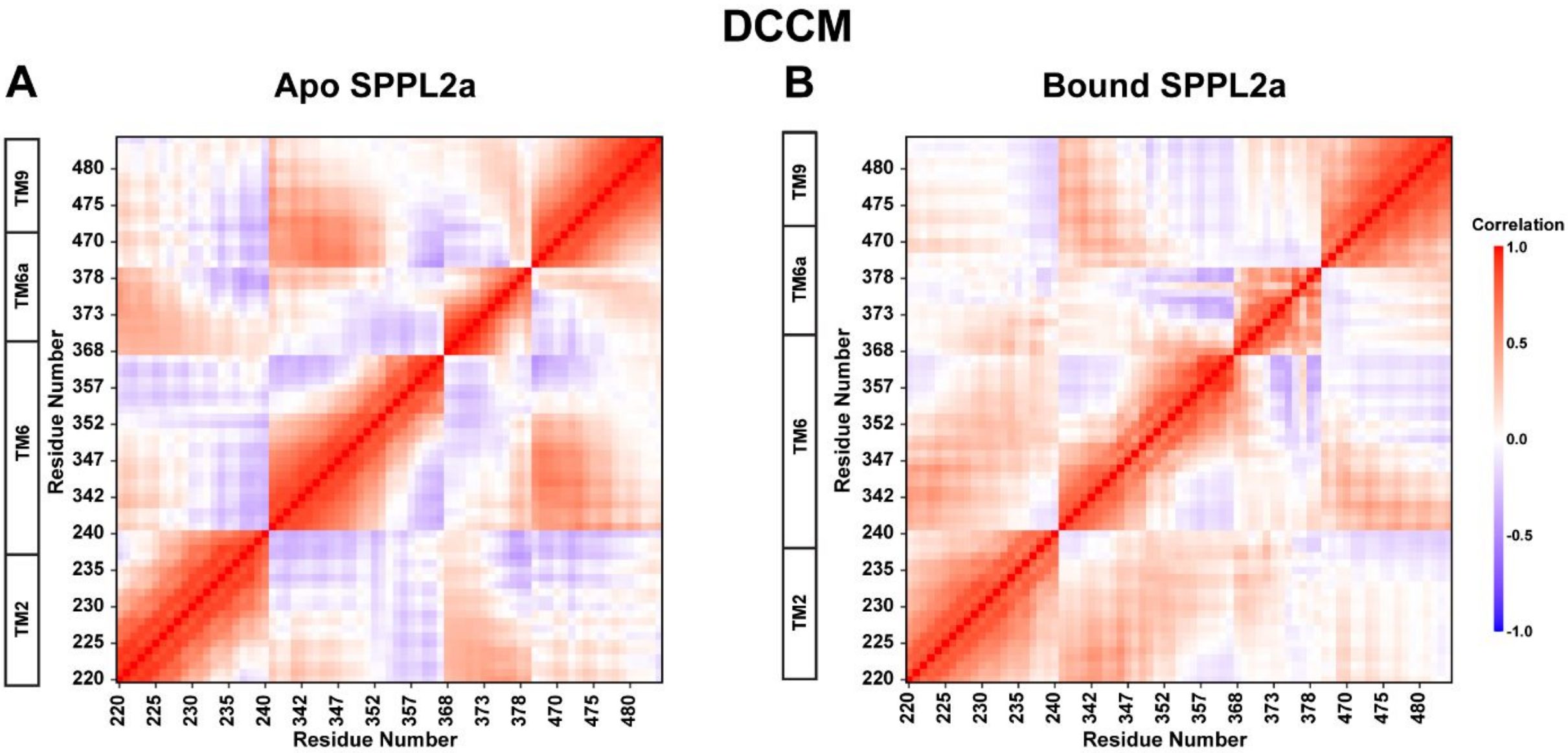

Figure S6. Comparative dynamic cross-correlation maps (DCCM) in intramembrane protease SPPL2a. Comparison of correlations between TM2, TM6, TM6a, TM9 across **(A)** apo SPPL2a and **(B)** bound SPPL2a.

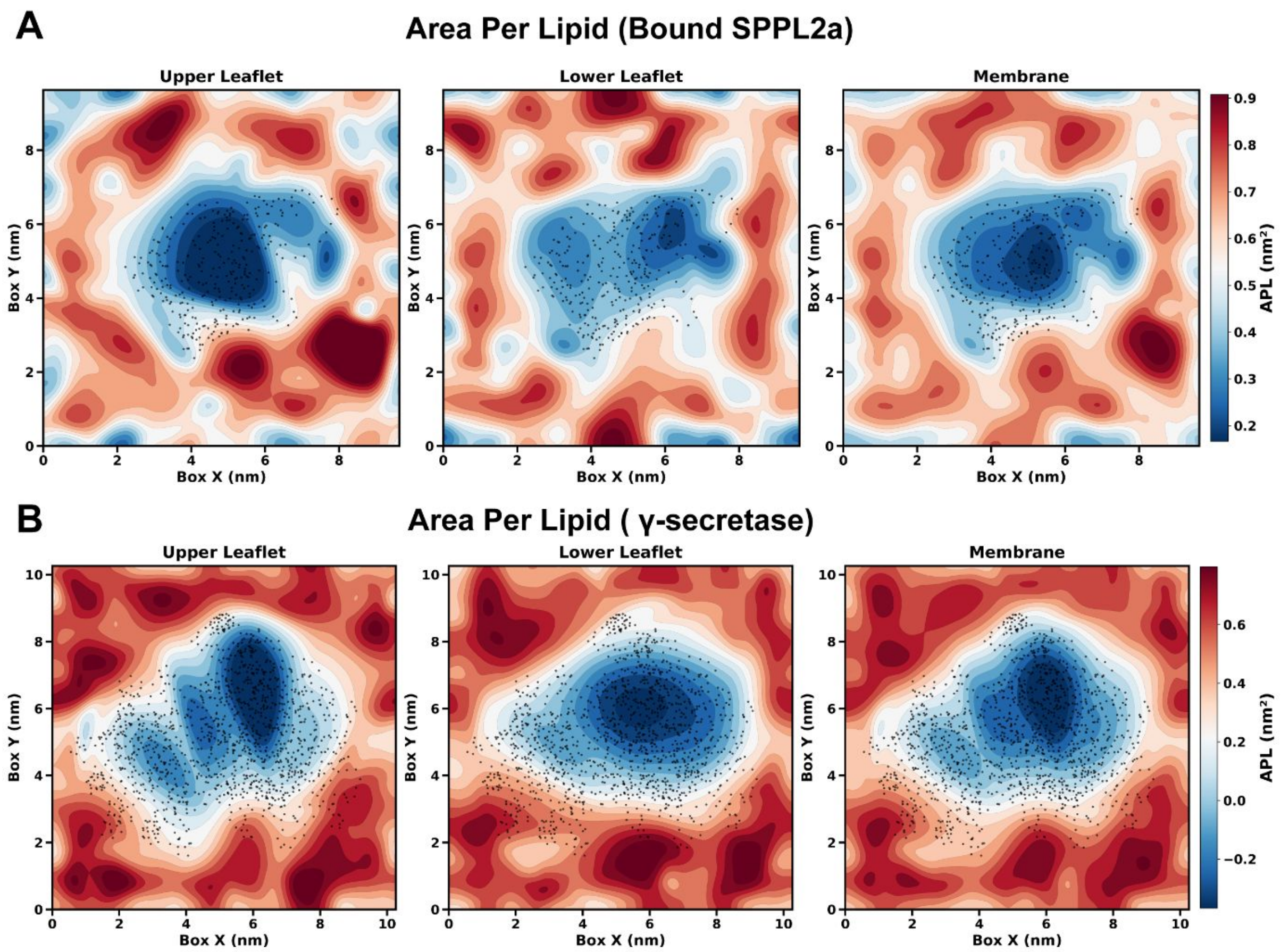

Figure S7. Area per Lipid plots of the intramembrane aspartyl protease systems. Contour plots demonstrating the local lipid organization around the protein-transmembrane interface of **(A)** inhibitor-bound SPPL2a and **(B)** γ-secretase with inhibitor-bound PS1.

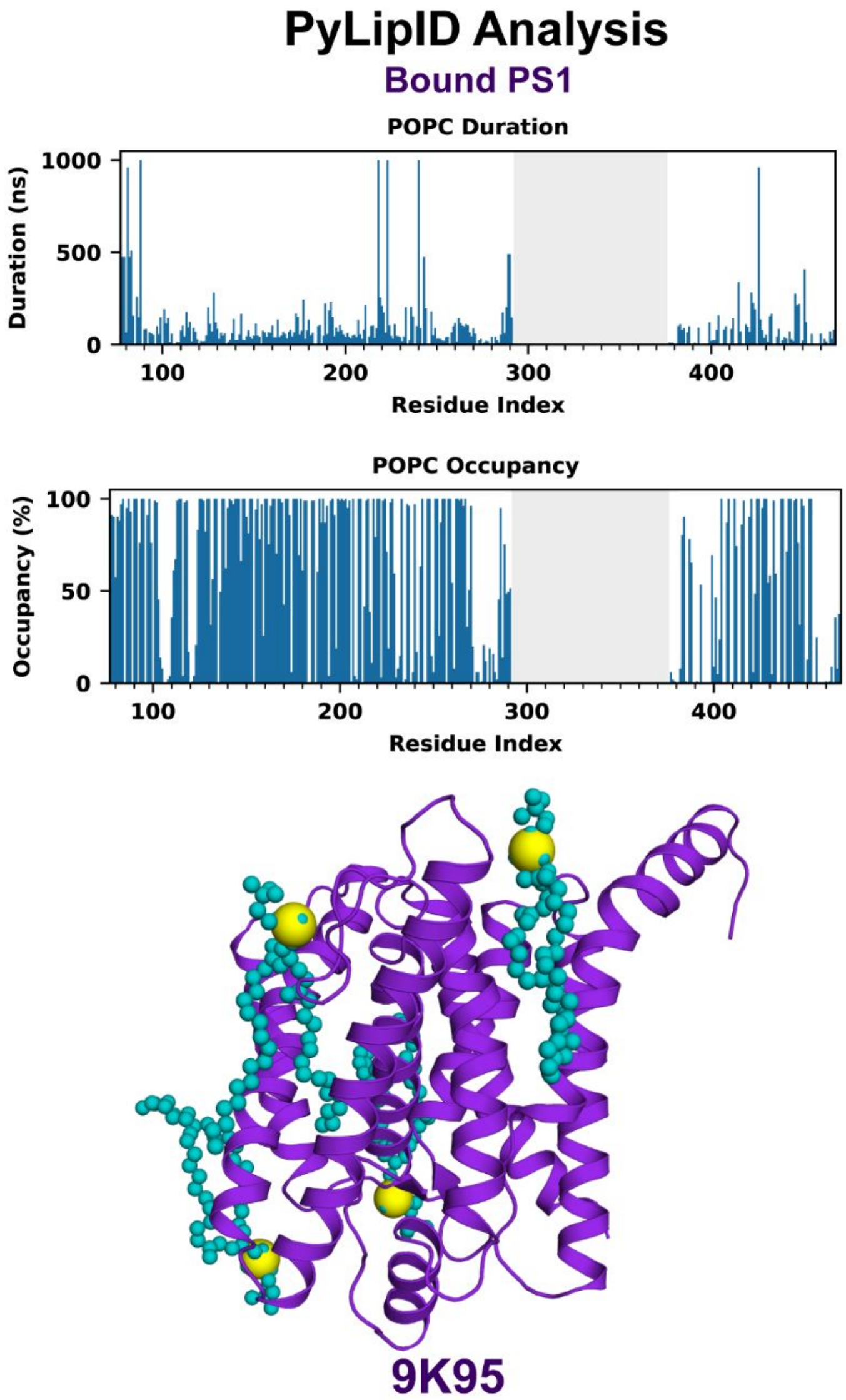

Figure S8. PyLipID analysis of site-specific lipid-protein interactions in bound PS1 subunit of the γ-secretase complex. The duration (residence time) and occupancy of the interactions are represented in bar plots, along with the structural representation illustrating the persistent binding locations of POPC lipids in bound PS1.

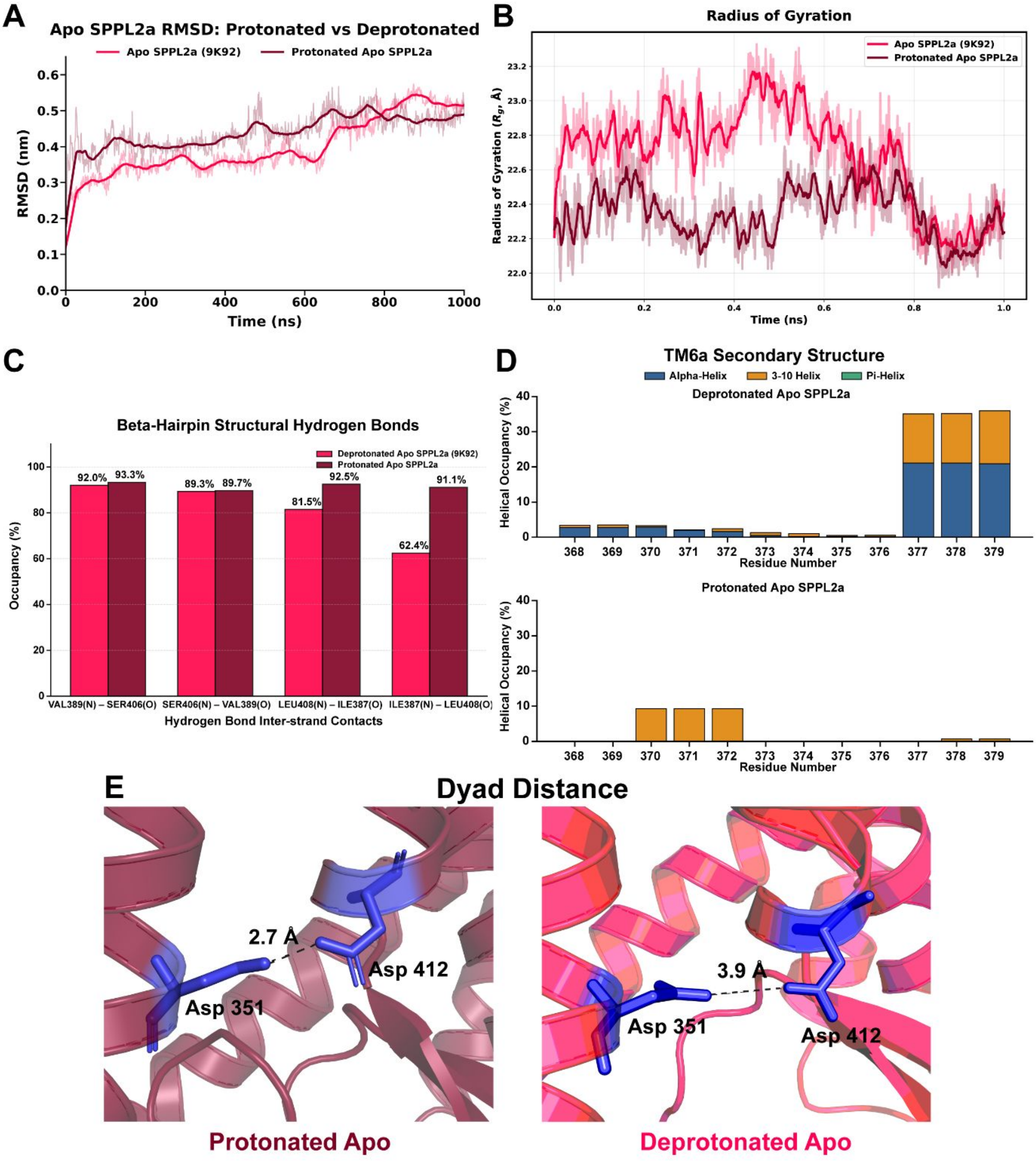

Figure S9. Comparative structural and conformational dynamics of apo SPPL2a across protonation states. (A) Root Mean Square Deviation of the protein backbone for protonated and deprotonated SPPL2a over 1 μs trajectory. **(B)** Radius of gyration analysis illustrating the global compactness of protonated and deprotonated apo SPPL2a**. (C)** Percentage occupancy comparison of the characteristic β-hairpin hydrogen-bonding network. **(D)** Secondary structure analysis of the TM6a region in protonated and deprotonated apo SPPL2a comparing helicity percentage. **(E)** Structural snapshots illustrating the distance between the catalytic Asp351 and Asp412 dyad residues.
